# Anti-Inflammatory and Antioxidant Properties of Dehydrated Potato-Derived Bioactive Compounds in Intestinal Cells

**DOI:** 10.3390/ijms20236087

**Published:** 2019-12-03

**Authors:** Manuela Giovanna Basilicata, Giacomo Pepe, Shara Francesca Rapa, Fabrizio Merciai, Carmine Ostacolo, Michele Manfra, Veronica Di Sarno, Giuseppina Autore, Daniela De Vita, Stefania Marzocco, Pietro Campiglia

**Affiliations:** 1 Department of Pharmacy, School of Pharmacy, University of Salerno, Via Giovanni Paolo II 132, I-84084 Fisciano, Italy; mbasilicata@unisa.it (M.G.B.); gipepe@unisa.it (G.P.); srapa@unisa.it (S.F.R.); fmerciai@unisa.it (F.M.); vdisarno@unisa.it (V.D.S.); autore@unisa.it (G.A.); 2 PhD Program in Drug Discovery and Development, University of Salerno, Via Giovanni Paolo II 132, I-84084 Fisciano, Italy; 3 Department of Pharmacy, University of Naples Federico II, Via D. Montesano 49, I-80131 Napoli, Italy; ostacolo@unina.it; 4 Department of Science, University of Basilicata, Viale dell’Ateneo Lucano 10, I-85100 Potenza, Italy; michele.manfra@unibas.it; 5 Department of Environmental Biology, University of Rome “La Sapienza”, I-00185 Rome, Italy; daniela.devita@uniroma1.it; 6 European Biomedical Research Institute of Salerno, Via De Renzi 50, I-84125 Salerno, Italy

**Keywords:** bioactive peptides, in vitro gastrointestinal digestion, intestinal epithelial cells, inflammation, oxidative stress, potato proteins

## Abstract

Inflammation and oxidative stress are always more recognized as responsible for chronic disease at the intestinal level. Currently, a growing interest is addressed to the discovery of diet-derived products which have anti-inflammatory and antioxidant properties. This work aims to characterize the pharmacological potential of dehydrated potatoes. For this purpose, a simulated gastrointestinal digestion was carried out. The bioaccessible peptides were fractionated on the basis of their molecular weight and tested on intestinal epithelial cells (IEC-6) under oxidative and inflammatory conditions. Our results demonstrate that the tested peptide fractions were able to significantly inhibit tumor necrosis factor-α release and cycloxygenase-2 and inducible nitric oxide synthase expression. The tested peptides also showed significant antioxidant activity, being able to both reduce reactive oxygen species (ROS) release, also from mitochondria, and nitrotyrosine formation, and increase the antioxidant response by heme oxygenase-1 and superoxide dismutase expression. Moreover, the peptide fractions were able to significantly increase the wound repair in IEC-6. The obtained results indicate the anti-inflammatory and antioxidant potential of dehydrated potatoes at the intestinal level.

## 1. Introduction

Inflammation and oxidative stress play a pivotal role in many chronic diseases, also affecting the gastrointestinal (GI) tract [1]. The main characteristic of the inflammatory cascade begins by infiltration of inflammatory cells into the mucosa and release of proinflammatory mediators such as cytokines, proinflammatory enzymes, chemokines, increased expression of adhesion molecules, and release of reactive oxygen species (ROS) [2,3]. ROS production is a key event in the progression of many inflammatory disorders, including those involving the GI tract. Oxidative stress is presently considered as a potentially critical mechanism in the pathogenesis, progression, and severity of inflammatory GI diseases [4]. Low physiological levels of ROS are indispensable for a plethora of signaling pathways [5,6]. However, excessive production of ROS results in a persistent oxidative stress condition, which causes tissue damage [7]. In the intestine, the interruption of the mucosal barrier, also induced by ROS, activates the innate immune system very quickly and results in an acute inflammatory response that begins in the lamina propria. Polymorphonuclear leukocytes migrate to the site of injury, engulf invading pathogens, and secrete ROS, granular enzymes, and vasoactive and proinflammatory mediators [8]. ROS production by these cells can create a hypoxic niche due to oxygen consumption, which may aid in the resolution of inflammation [9]. In particular, deregulation of the mitochondrial electron transport chain, with increased mitochondrial ROS (mtROS) levels, was observed in inflammatory bowel disease (IBD) patients and decreasing mtROS ameliorated colitis [10].

The integrity of the epithelial barrier is an essential prerequisite for intestinal homeostasis. Evidence indicates that the disruption of the intestinal barrier, and the increased paracellular permeability, play a crucial role in the pathogenesis of GI diseases, such IBD, alcoholic endotoxemia, infectious enterocolitis, celiac disease, and necrotizing enterocolitis [11]. In detail, a variety of oxidative stress and inflammatory mediators, such as ROS and cytokines, disrupt the intestinal barrier integrity. In these cases, the capacity to rapidly reseal the epithelial layer is critical to avoid or minimize the exposure of immune cells to microbiota, which would lead to the initiation and perpetuation of an inflammatory and oxidative stress response.

Thus, the consequences of altered ROS levels on multifactorial GI inflammation are still not well understood, but the importance of maintaining the redox balance is strongly emerging.

In particular, dietary antioxidants can supplement the antioxidant system and help to reduce the degenerative oxidative damage [12]. Vegetable matrices represent important sources of several classes of antioxidant compounds such as polyphenols and bioactive peptides, which are often used as ingredients for developing functional foods and nutraceutical products [13,14]. 

Among them, potatoes (*Solanum tuberosum*) are of particular interest because, in terms of production, they are the fourth most produced crop after rice, wheat, and corn [15] and in terms of consumption, they have been a staple food in many traditional diets of the Western world. Potatoes are often considered nutritionally beneficial for their high starch content (75% of the total dry matter), which supplies the body with energy, and for the presence of macronutrients such as dietary fiber and many proteins, and other important micronutrients such as several vitamins, polyphenolic compounds, and minerals [16]. All these elements are associated with a decreased risk of morbidity and mortality [17].

Despite their low protein concentration of 1.7%, potatoes are the second largest protein-supplying crop per hectare grown after wheat [18]. Potato proteins have been proven to be nutritionally superior to most other plant and cereal proteins and relatively close to egg protein [19]. Increasing evidence has indicated that potatoes, as a rich source of bioactive compounds, exhibit health-promoting properties, including antioxidant, anti-inflammatory, and anticancer activity [20]. The production of short-chain fatty acids (SCFAs) by bacterial fermentation of potato fibers (PFs) has been associated with prolonging remission of IBD by modulating the immune response in various cell types [21]. It has been also reported that PFs are fermentable and anti-inflammatory during colitis in mice. From a clinical perspective, dietary PFs delayed body weight loss induced by colitis, which would be synonymous to prolonging remission phases of ulcerative colitis; this effect was associated with SCFA intestinal concentrations. Generally, it is believed that the anti-inflammatory properties of potato are also related to proteins with protease inhibitor activity. At the GI level, potato proteins have been demonstrated to alleviate perianal inflammation by inhibiting fecal proteases [22]. On the other hand, the anti-inflammatory effect of potato is suggested to be attributable to the antioxidant content [23]. Human cohort studies proved the systemic anti-inflammatory effect of potato as measured by serum C-reactive protein, at a level that was inversely correlated with the serum concentration of certain potato antioxidants [24]. Potato proteins have high nutritional value, attributed to a high proportion of the essential amino acids such as lysine [15], threonine, and tryptophan, and relatively high proportions of sulfur-containing amino acids such as methionine and cysteine [18]. Potato proteins are often classified into three groups, namely, patatin, protease inhibitors, and high-molecular-weight proteins. These high-quality proteins are able to exert beneficial effects on human health such as lower allergic response [25], antimicrobial effects [26], antioxidant potential, the regulation of blood pressure and blood serum cholesterol control [27,28], and anticancer properties [29]. 

On the other hand, despite the positive functional and nutritional properties, potatoes represent a contradictory food. To date, more than half of all potatoes consumed are chips, fried and roasted potatoes, or processed potato products, especially among older children and young adults [30]. The frequent consumption of fried potatoes appears to be associated with developing obesity, diabetes, and cardiovascular disease (CVD) due to their large starch content and high glycemic index [31].

For these reasons, in order to maintain the beneficial properties of potatoes and decrease the higher intakes of trans fatty acids, oxidized lipids, acrolein, acrylamide, furan, and glycidamide of fried potatoes, in this work the antioxidant, anti-inflammatory, and wound-healing repair properties of dehydrated potatoes were investigated. In detail, the intestinal protection of bioactive peptides released during GI digestion of dehydrated potatoes was evaluated in rat intestinal epithelial cell line (IEC-6) stimulated by *E. coli* lipopolysaccharide (LPS) plus interferon-γ (IFN).

## 2. Results

### 2.1. Simulated GI Digestion of Dehydrated Potatoes

**Figure 1:**
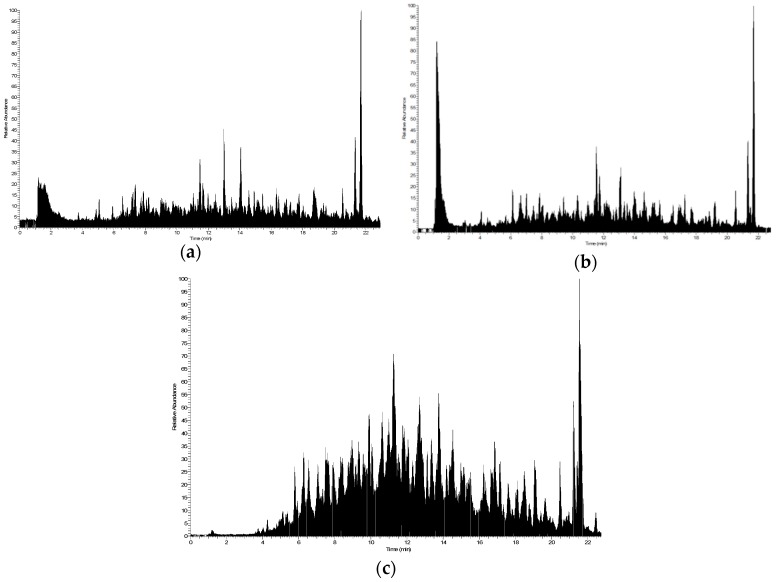
Total ion current (TIC) chromatograms of peptides derived from simulated GI digestion of dehydrated potatoes. The peptide fractions were obtained by ultrafiltration with different cut-off membranes (**a**): 3–10 kDa; (**b**): 1–3 kDa; (**c**): <1 kDa).

In vitro GI digestion of dehydrated potatoes was carried out. During the oral, gastric, and intestinal digestion steps, the potato parent proteins were denatured and hydrolyzed by the action of proteolytic enzymes releasing peptides with different molecular weights. In order to simplify this highly complex matrix, GI digest was fractionated by ultrafiltration using centrifugal filter devices with different NMWL (Nominal Molecular Weight Limit) obtaining three peptide aliquots: 3–10, 1–3, and <1 kDa. Peptide fractions of intestinal digesta were monitored by liquid chromatography-high resolution mass spectrometry (LC-HRMS) (Figure 1). The complete list of peptides identified in the three fractions are reported in Supplementary Material File (Tables S1–S3). LC-MS/MS analysis allowed the identification of 590 bioaccessible peptides, with 245 peptides belonging to the 3–10 kDa fraction, 140 to the 1–3 kDa fraction, and 205 to the <1 kDa fraction. The bioaccessible peptides, released during GI digestion of dehydrated potatoes, belong to two major protein groups: patatin and tuberinin. Patatin, also known as tuberin, is an important family of glycoproteins and represents approximately 40% of the soluble protein. Similarly, tuberinin represents 30–40% of the total tuber protein and includes protease inhibitor I, potato aspartate protease inhibitor, potato cysteine protease inhibitor, potato Kunitz-type protease inhibitor, and other serine protease inhibitors [22,23].

### 2.2. Three Fractions of Dehydrated Chips Peptides Did Not Affect IEC-6 Viability

To elucidate the influence of three fractions on viability of IEC-6 under our experimental conditions, cells were treated with three different fractions (in the range 1–10 µg/mL) for 24 h. Our data indicated that viability of IECs was not affected by the peptides (data not shown).

### 2.3. Peptide Fractions Reduced TNF-α Release

**Figure 2:**
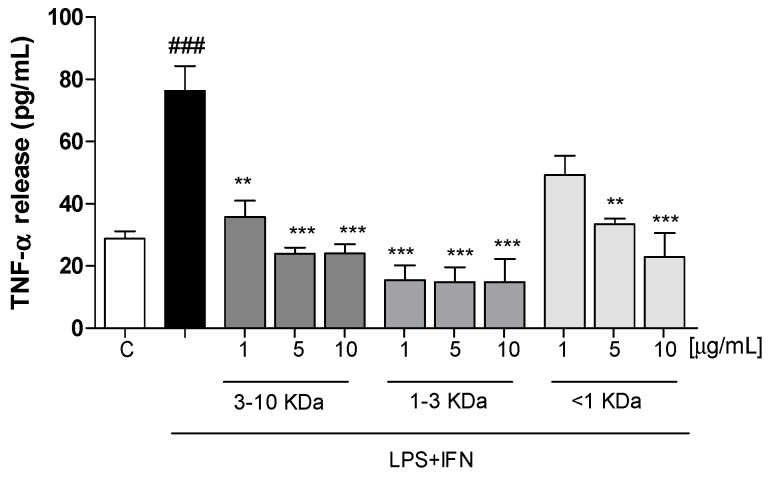
Effect of three dehydrated potato peptides (1–10 µg/mL) on TNF-α release, induced by LPS + IFN in IEC-6 cellular medium, evaluated by ELISA assay. The figure shows that the three tested fractions significantly inhibited TNF-α release. Data are expressed as pg/mL of TNF-α release. C denotes control group. *** and ** denote respectively *p* < 0.001 and *p* < 0.01 vs. LPS + IFN; ### denotes *p* < 0.001 vs. C.

The effect of three fractions on TNF-α levels in IEC-6 cellular medium was evaluated using an ELISA assay. Our results showed that the tested peptides (1–10 µg/mL) significantly inhibited TNF-α release, induced by LPS + IFN, from IEC-6 cells into the medium (*p* < 0.01 vs. LPS + IFN; Figure 2). This effect was observed for the fractions 3–10 KDa and 1–3 KDa at all tested concentrations and for the fraction <1 KDa at the concentrations of 10 and 5 µg/mL.

### 2.4. Peptides of Dehydrated Chips Reduced Cycloxygenase-2 (COX-2) and Inducible Nitric Oxide Synthase (iNOS) Expression in LPS + IFN-Stimulated IEC-6

**Figure 3:**
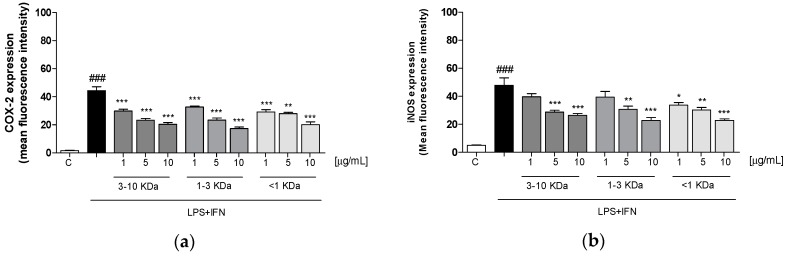
Effect of the three fractions of dehydrated potato peptides (1–10 µg/mL) on LPS + IFN-stimulated IEC-6 cells. The figure shows that the three tested fractions significantly inhibited COX-2 (*p* < 0.01 vs. LPS + IFN) (**a**) and iNOS (*p* < 0.05 vs. LPS + IFN) (**b**) expression, evaluated by the cytofluorimetric technique. Values are expressed as mean ± SEM of mean fluorescence intensity. Comparisons were performed using a one-way analysis of variance and multiple comparisons were made by Bonferroni’s post-test. ***, **, and * indicate respectively *p* < 0.001, *p* < 0.01, and *p* < 0.05 vs. LPS + IFN. ### denotes *p* < 0.001 vs. C.

In order to analyze the anti-inflammatory potential of the tested peptides, we evaluated the expression of enzymes mainly involved in inflammatory reactions, such as COX-2 and iNOS, by the cytofluorimetric technique. Our results showed that three fractions (1–10 µg/mL) inhibited COX-2 expression in IEC-6 cells at all tested concentrations. The inhibitory effect on iNOS expression was exerted by all the peptides at the two higher concentrations, except for the fractions 3–10 KDa and 1–3 KDa, which inhibited iNOS only at the highest tested concentrations (*p* < 0.05 vs. LPS + IFN; Figure 3a,b).

### 2.5. Peptide fractions Reduced Intracellular ROS Release, Mitochondrial Superoxide Production, and Nitrotyrosine Formation

**Figure 4:**
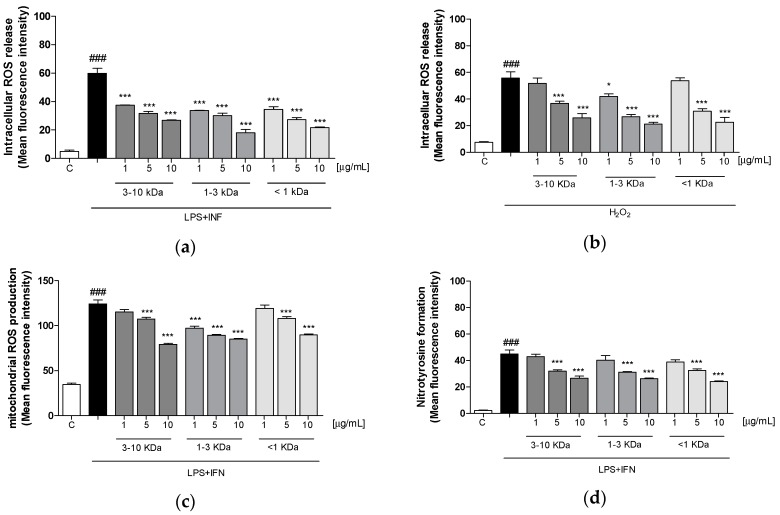
Effect of the three fractions on intracellular and mitochondrial ROS release and on nitrotyrosine formation. The three tested fractions (1–10 µg/mL) significantly inhibited intracellular ROS release, both (**a**) in LPS + IFN-stimulated cells and (**b**) in cells treated with H_2_O_2_ (*p* < 0.001 vs. LPS + IFN; *p* < 0.05 vs. H_2_O_2_), evaluated by means of the probe 2′,7′ dichlorofluorescein-diacetate (H_2_DCF-DA). (**c**) Mitochondrial superoxide production (*p* < 0.001 vs. LPS + IFN) was evaluated by MitoSOX Red. (**d**) Reduction of nitrotyrosine formation by the fractions (1–10 µg/mL) (*p* < 0.001 vs. LPS + IFN) in LPS + IFN-stimulated cells. Values are expressed as mean ± SEM of mean fluorescence intensity, evaluated by the cytofluorimetric technique. Comparisons were performed using a one-way analysis of variance and multiple comparisons were made by Bonferroni’s post-test. *** and * denote respectively *p* < 0.001 and *p* < 0.05 vs. LPS + IFN or vs. H_2_O_2_. ### denotes *p* < 0.001 vs. C.

The antioxidant potential of three fractions was evaluated by measuring the intracellular ROS production in LPS + IFN-stimulated IEC-6 cells. It was found that the peptides (1–10 μg/mL) significantly and in a concentration-related manner inhibited ROS production in IEC-6 cells (*p* < 0.001 vs. LPS + IFN; Figure 4a). To further evaluate their antioxidant potential, the peptides (1–10 μg/mL) were tested in IEC-6 cells treated with the pro-oxidant stimulus H_2_O_2_ (1 mM). Our results showed that three fractions (1–10 µg/mL) significantly inhibited ROS production in IEC-6 cells (*p* < 0.05 vs. H_2_O_2_; Figure 4b), except for the fraction <1 KDa, which was significantly effective only at the two highest concentrations.

We also measured mitochondrial superoxides by means of flow cytometry. As shown in Figure 4c after 15 min, LPS + IFN-induced superoxide release from mitochondria resulted in being significantly reduced by peptide fractions (1–10 µg/mL; *p* < 0.001 vs. LPS + IFN; Figure 4c). Moreover, we evaluated the formation of nitrosative stress markers, such as nitrotyrosine, in LPS + IFN-stimulated IEC-6 cells. As shown in Figure 4d, three fractions (1–10 µg/mL) significantly reduced nitrotyrosine formation in the cells (*p* < 0.001 vs. LPS + IFN; Figure 4d).

### 2.6. Peptide Fractions Increased HO-1 and SOD Expression in LPS + IFN-Stimulated IEC-6

**Figure 5:**
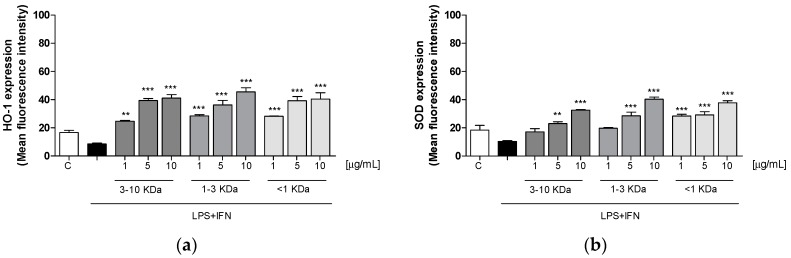
Effect of the three fractions (1–10 µg/mL) on HO-1 (**a**) and SOD (**b**) expression in LPS + IFN-stimulated IEC-6 cells. The three tested fractions significantly increased HO-1 (*p* < 0.01 vs. LPS + IFN) (**a**) and SOD (*p* < 0.01 vs. LPS + IFN) (**b**) expression, evaluated by the cytofluorimetric technique. Values are expressed as mean ± SEM of mean fluorescence intensity. Comparisons were performed using a one-way analysis of variance and multiple comparisons were made by Bonferroni’s post-test. *** and ** indicate respectively *p* < 0.001 and *p* < 0.01 vs. LPS + IFN.

Oxidative stress is due to a disequilibrium between pro-oxidant and antioxidant factors. Expression of cytoprotective and antioxidant enzymes, such as HO-1 and SOD, was significantly increased by three fractions (1–10 µg/mL) in LPS + IFN-stimulated IEC-6 cells (*p* < 0.01 vs. LPS + IFN; Figure 5a,b).

### 2.7. Effect of Peptide Fraction on IEC-6 Cellular Migration

**Figure 6:**
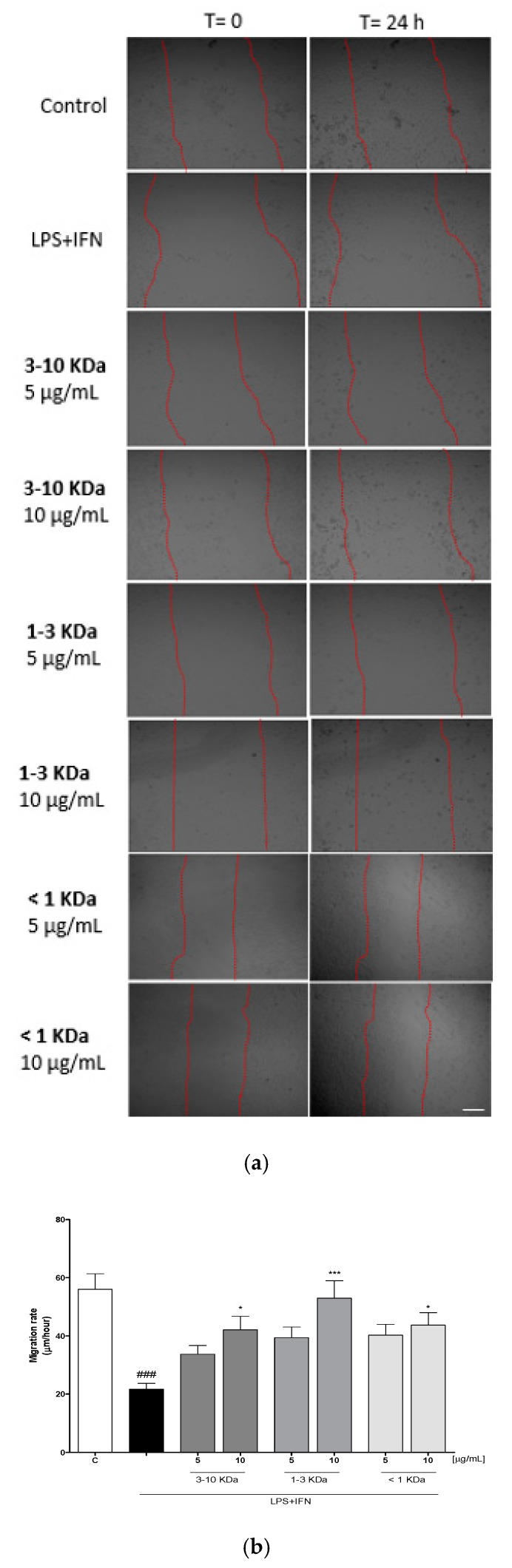
Effect of the three fractions (5–10 µg/mL) on cell migration after induction of mechanical scratch in IEC-6 treated with LPS + IFN; Bar = 150µm (**a**), and the quantitative analysis expressed as IEC-6 migration rate after 24 h (**b**). Values are expressed as migration rate (µm/hour). *** and * denote respectively *p* < 0.001 and *p* < 0.05 vs. LPS + IFN. ### denotes *p* < 0.001 vs. C.

In order to assess the effect of the tested peptide fractions on the reconstitution process at the intestinal level, we carried out a wound-healing assay, to evaluate cellular migration, on treated IEC-6 monolayers. On complete confluence, a wound was created in IEC-6 monolayers by scraping and time-lapse video microscopy was used to monitor cellular migration at the wound site for 24 h. Different cells were selected and their migration distances were measured at different time points. Figure 6 shows a significant increase of the cellular migration speed of IEC-6 cells treated with graded concentrations of peptide fractions of dehydrated chips (5–10 µg/mL) compared to LPS + IFN-treated cells (*p* < 0.05 vs. LPS + IFN; Figure 6a,b).

## 3. Discussion

White potatoes have been a staple food in many traditional diets of the Western world. In recent years, the overall consumption of potatoes has declined, but processed potato intake (e.g., French fries and chips) has dramatically increased. Consumption of processed potato has been associated with an increased risk of developing several pathologies such as obesity, diabetes, and CVD [32]. However, compared with other common carbohydrate sources, potatoes are characterized by high water content and consequently have a low energy density [33]. In addition, potatoes provide other important micronutrients, which are all associated with beneficial health effects [17]. In this context, in order to preserve the beneficial effect of potatoes and to decrease the detrimental effects of processed potatoes, we have investigated the antioxidant and anti-inflammatory properties of white potatoes obtained by the dehydration process. Dehydration is a cooking technique that preserves food for indefinite periods by extracting the moisture, thereby inhibiting the growth of microorganisms and preserving the biological properties of potatoes. In the present work, we have evaluated the bioaccessible peptides released during simulated GI digestion of dehydrated potatoes. The peptides were fractionated on the basis of their molecular weight, characterized by LC-HRMS experiments, and tested on intestinal epithelial cells under oxidative and inflammatory conditions. The obtained results indicated that all the three peptide fractions of dehydrated potatoes possess anti-inflammatory and antioxidant activity and promote cell migration in the IEC-6 cells. LPS-induced release of cytokines could contribute to the deterioration of intestinal inflammation. In particular, studies have revealed that intestinal epithelial cells could release inflammatory cytokines with LPS plus IFN-γ stimulation [34]. One of the major cytokines playing a pivotal role in inflammatory GI diseases is TNF-α and it resulted in being significantly upregulated by proinflammatory stimuli in IECs [35]. Also, proinflammatory enzymes such as iNOS or COX-2 caused by intestinal inflammation are found higher in the inflammatory pathologies of the GI tract and contribute both to the amplification of the inflammatory response (also via TNF-α) and to the oxidative stress status [36,37]. In our experiments, the three fractions significantly inhibited TNF-α release at all concentrations, as well as COX-2 and iNOS expression at the higher tested concentrations. Inflammatory response is characterized by the production of highly reactive intermediates as ROS. On the other hand, the same ROS are able to promote the inflammatory cascade acting on transcription factors and cytokines production [38,39]. Our results indicated that the three fractions were able to inhibit ROS release both during inflammation and in oxidative stress conditions. The current literature supports a key correlation between mitochondrial function and intestinal inflammation. Recent studies have proposed mitochondria as significant cellular drivers and mediators of the inflammatory process. It seems likely that mitochondrial ROS production plays a key role in the intestinal inflammation associated with inflammatory pathologies of the GI tract, such as IBD [40]. Furthermore, treatment with a mitochondria-targeted antioxidant, MitQ, reduced mitochondrial ROS and protected against experimental colitis in mice subjected to dextran sodium sulfate [10]. This evidence further supports the potential of the tested potato peptides considering that these peptides induced an inhibition of superoxide release from mitochondia, as assessed by MitoSOX Red. ROS are able to interact with NO to form nitrotyrosine, a marker of nitrosative stress, which induces protein function alterations and tissue damage. Nitrotyrosine formation in IEC-6 cells was significantly reduced by the peptides during inflammatory response. On the other hand, here we also report that the peptide fractions enhance the antioxidant cellular response by promoting the production of Nrf2-regulated cytoprotective enzymes, such as HO-1, and SOD. This is in sharp contrast with the pro-oxidant effect reported for heat-processed potatoes, particularly fried, which contain high levels of acrylamide, able to increase ROS production and nitrosative stress as well as decrease the endogenous levels of cytoprotective enzymes [41,42]. One of the most important functions of the intestinal enterocytes is to create a protective barrier; the intactness of the IECs monolayer as well as the IECs’ function during the restitution process are of primary importance to elude the initiation and the perpetuation of inflammatory and oxidative stress response. Generally, during inflammatory events, this barrier is damaged, and the healing processes are of pivotal importance to restore preinflammatory conditions, avoiding potential damage to the entire organism [11]. The results on cell migration indicate that the tested peptides significantly increased cellular migration speed in IEC-6, thus improving the restitution process and supporting the intactness of the cellular monolayer. This effect could be also related to the antioxidant properties of the tested peptides, considering the pivotal effect of ROS in impairing the intestinal barrier homeostasis. Although this study was performed in an experimental in vitro model, on IEC, and needs further investigation, it demonstrated that all the three dehydrated potato-derived peptide fractions have a significant anti-inflammatory and antioxidant potential, also promoting IEC-6 migration. These findings highlight the pharmacological potential of these peptides in IBD.

## 4. Materials and Methods 

### 4.1. In Vitro GI Digestion of Dehydrated Potatoes, Fractionation, and Identification of Released Peptides

Dehydrated chips samples were kindly donated by Felix S.r.l. (Battipaglia, SA, Campania, Italy) and prepared as follows: potatoes were peeled and sliced before being washed with fresh water (3–5 times) to remove the external starch; the washing stage was repeated until clear and transparent water was obtained; after drying, potato slices were baked under ventilation at 85 °C for 90 min, giving, upon cooling, the dehydrated chips. 

The simulated GI digestion of potato products was performed according to the protocol reported by Pepe et al. [39]. GI digestion was distinguished into salivary, gastric, and duodenal digestive steps. Briefly, 15 g samples were mixed with artificial saliva and homogenized for 3 min. Then, the mixture was incubated with pepsin at 37 °C for 2 h to pH = 2 and the reaction was stopped by heating the solution. The gastric digest was incubated with pancreatin, chymotrypsin, and bile salts at 37 °C for 2 h to pH 7.5 and the reaction was stopped, bringing the solution to pH 2. Finally, the mixture was centrifuged at 6000 rpm at 4 °C for 10 min (Mikro 220R centrifuge, Hettich, Germany), filtered on 0.45 µm filters (Phenex RC membrane, Phenomenex, Bologna, Italy), lyophilized (LyoQuest-55, Telstar Technologies, Spain), and stored at −80 °C. 

The GI digest was fractionated by ultrafiltration with different cut-off membranes to obtain three peptide fractions with different molecular weight. In detail, a preliminary filtration was carried out for the intestinal digest (2 mg mL^−1^) using filters with 10,000 NMWL (Amicon^®^ Ultra-4 10K, Merck Millipore, Tullagreen, Ireland). The devices were centrifuged for 25 min at 6000 rpm at 25 °C. Subsequently, the permeate was loaded on filter devices with 3000 NMWL (Amicon^®^ Ultra-4 3K) and centrifuged for 60 min at 6000 rpm at room temperature. The retentate obtained was the fraction I (3–10 KDa). In contrast, 4 mL of eluate were loaded on a Microsep Advance Centrifugal Device 1 K (Pall Corporation, Ann Arbor, MI, USA) and were centrifuged for 90 min at 6000 rpm [38]. The retentate was the fraction II (1–3 KDa) while the permeate obtained mainly was the fraction III (<1 KDa), composed of peptides with medium and low molecular weight, respectively. After lyophilization, the peptide fraction III was solubilized in distilled water and loaded on a Strata-X 33µm Polymeric Reversed Phase SPE cartridge (Phenomenex^®^), previously equilibrated in distilled water, then eluted with MeOH 2% *v*/*v* formic acid.

Peptide fractions were monitored by LC-MS/MS according to the protocol previously reported by Basilicata et al. 2018 with slight modifications [43]. HRMS experiments were performed on an LTQ Orbitrap XL mass spectrometer (Thermo Scientific, Bremen, Germany) through an electrospray source. MS parameters were set as follows: spray voltage was set at +3.5 kV; sheath gas, 30 (arbitrary units); auxiliary gas, 10 (arbitrary units); capillary temperature, 250 °C. Data-dependent mode MS/MS was performed over the *m*/*z* range of 300–2000, at 30,000 resolution. MS/MS spectra collection parameters were: collision energy, 35%; isolation window, 2 *m*/*z*; minimum signal threshold, 150; monoisotopic precursor, enabled. Ion trap and Orbitrap ion injection times were set to 50 and 100 ms, respectively. Raw MS/MS data files were converted to mzXML format, and a free trial of PEAKS 8.5 software (Bioinformatics Solutions Inc., Waterloo, Canada) was employed for peptide sequence determination. Search was performed using a database search tool, by searching against Swiss-Prot/UniProt database (Release 2015_11) taxonomy *Solanum tuberosum*, with an improved algorithm that validates and assists the database search with de novo sequencing results with the following settings: enzymes: pepsin, trypsin, chimotrypsin; peptide charges from +1 to +3, monoisotopic precursor mass; fragmentation mode, CID (y and b ions); precursor mass tolerance, 15 ppm; fragment mass tolerance of 0.5 Da.

### 4.2. Biological Assays

#### 4.2.1. Cell Culture and Treatment

IEC-6 cells (CRL-1592) were purchased from the American Type Culture Collection (ATCC, Rockville, MD, USA). These cells, derived from normal rat intestinal crypt cells, were grown in Dulbecco’s modified Eagle’s medium (DMEM, 4 g/L glucose) with 10% (*v*/*v*) fetal bovine serum (FBS), 2 mM l-glutamine, 1.5 g/L NaHCO_3_, and 0.1 unit/mL bovine insulin. Cells between the 17th and 20th passages were used for these experiments. IEC-6 cells were plated and, after adhesion, treated with the three fractions of dehydrated potato peptides (3–10 kDa, 1–3 kDa, and <1 kDa; 10–1 μg/mL) for 1 h alone and then in the presence of lipopolysaccharides from *E. coli* (LPS; 10 μg/mL) plus interferon-γ (IFN; 10 U/mL) [44,45] for different experimental times, as outlined below. 

#### 4.2.2. Antiproliferative Activity

IEC-6 cells (2 × 10^3^ cells/well) were plated on 96-well plates and allowed to adhere for 24 h at 37 °C in a 5% CO_2_ atmosphere. Thereafter, the medium was substituted with either a new one alone or one containing serial dilutions of the three fractions (1–10 μg/mL) and incubated for 24 h. The antiproliferative activity was evaluated using the colorimetric assay of 3-(4,5-dimethylthiazol-2-yl)-2,5-diphenyltetrazolium bromide (MTT), as formerly reported [46]. MTT (5 mg/mL) was then added to IEC-6 cells. After 3 h, cells were lysed with 100 µL of a solution containing 50% (*v*/*v*) N,N-dimethylformamide and 20% (*w*/*v*) sodium dodecyl sulphate (SDS; pH = 4.5). A microplate spectrophotometer reader (Titertek Multiskan MCC/340-DASIT, Cornaredo, Milan, Italy) was used to measure the optical density (OD) of released formazan in each well. The antiproliferative activity was measured as: % dead cells = 100 − [(OD treated/OD control) × 100].

#### 4.2.3. Tumor Necrosis Factor Determination

The TNF-α levels were assessed with an Enzyme-Linked Immuno Sorbent Assay (ELISA). IEC-6 cells were plated into 24-well plates (8 × 10^4^ cells/well) and allowed to adhere for 24 h. Cells were then treated with peptide fractions (1–10 μg/mL), as previously indicated, for 24 h. Supernatants from IEC-6 cells were then collected and a commercial kit (e-Bioscience, San Diego, CA, USA) was used to perform the ELISA, according to the manufacturer’s instructions (e-Biosciences, San Diego, CA, USA). Results were expressed as pg/mL as previously reported [47].

#### 4.2.4. Measurement of COX-2, iNOS, HO-1, and SOD Expression and Nitrotyrosine Formation by Cytofluorimetry

IEC-6 cells were plated into 96-well plates (2 × 10^3^ cells/well) and treated with the peptides in inflammatory conditions, as previously described, for 24 h in order to evaluate COX-2, iNOS, HO-1, and SOD expression and nitrotyrosine formation. For this analysis, the cells were collected and washed with phosphate-buffered saline (PBS). Fixing solution was added to cells for 20 min and then incubated in fix/perm solution for a further 30 min. Anti-COX-2 (BD Transduction Laboratories, Milan, Italy), anti-iNOS (BD Transduction Laboratories, Milan, Italy), anti-HO-1 (Santa Cruz Biotechnologies, Dallas, TX, USA), anti-SOD (Santa Cruz Biotechnologies, Dallas, TX, USA), and anti-nitrotyrosine (Merck Millipore, Milan, Italy) antibodies were then added for 1 h. The secondary antibody, in fixing solution, was added to the cells and cell fluorescence was then evaluated by a fluorescence-activated cell sorter (FACSscan; Becton Dickinson, Milan, Italy) and analyzed by Cell Quest software (version 4; Becton Dickinson, Milan, Italy) [35].

#### 4.2.5. Intracellular ROS Release Measurement and Mitochondrial Superoxide Evaluation with MitoSOX Red

ROS intracellular production was evaluated by the probe 2′,7′-dichlorofluorescein-diacetate (H_2_DCF-DA). The IEC-6 cells were seeded in 24-well plates (8 × 10^4^ cells/well) and allowed to adhere for one day. After adhesion, cells were incubated with three fractions (1–10 μg/mL) alone for 1 h and then coexposed to the tested compounds and LPS (10 μg/mL) plus IFN (10 U/mL) for a further 24 h. In another set of experiments, cells were treated with three fractions (1–10 μg/mL) alone for 1 h and then exposed simultaneously to the peptides and hydrogen peroxide (H_2_O_2_; 1 mM) for 1 h more. After the treatment, cells were collected, washed with PBS, and then incubated in PBS containing H_2_DCF-DA (10 μM). Cell fluorescence was evaluated after 15 min at 37 °C, using a fluorescence-activated cell sorter (FACSscan; Becton Dickinson, Franklin Lakes, NJ, USA), and was analyzed by Cell Quest software version 4 (Becton Dickinson, Milan, Italy), as previously reported [48]. 

In order to detect superoxide release from mitochondria in some experiments after cell treatment, as previously described, MitoSOX Red was used. MitoSOX Red (2.5 µM) was added for 10 min before fluorescence evaluation by means of flow cytometry. This indicator is a fluorogenic dye for highly selective detection of superoxide in the mitochondria of live cells and, once targeted to the mitochondria, it is oxidized by superoxide and exhibits red fluorescence. MitoSOX is rapidly oxidized by superoxide but not by other ROS-generating systems.

#### 4.2.6. Scratch Assay for Cellular Migration

Cell migration has been evaluated by using the scratch assay. This is a laboratory technique used to study cell migration and cell–cell interaction after making a scratch on a cell monolayer and capturing images at regular intervals by time-lapse microscope [49,50]. This assay has been used with multiple cell types and, as the monolayers close the scratch in a characteristic manner, it has been applied also to study cell polarization, matrix remodeling, cell migration, and numerous other processes [51]. In order to evaluate IEC-6 cellular migration, treated with three fractions (5–10 μg/mL), a wound-healing assay was performed, as previously reported [47]. IEC-6 cells (1 × 10^5^ cells/well, 24-well plates) were allowed to adhere for 24 h. A mechanical scratch was induced at the center of the IEC-6 monolayer by scraping cells with a sterile plastic p10 pipette tip. Cells were then washed with PBS and treated with three fractions (5–10 μg/mL) and LPS (10 μg/mL) plus IFN (10 U/mL) for 24 h. After the scratch, IEC-6 cells were then placed in a humidified and equilibrated (5% *v*/*v* CO_2_) incubation chamber of an Integrated Live Cell Workstation Leica AF-6000 LX at 37 °C for 24 h. A 10X phase contrast objective was used in order to record cell movements, with a frequency of acquisition of 10 min. To determine the migration rate of individual cells, we considered the distances covered from the initial time to the selected time points (bar of distance tool, Leica AF software, 2.3.5 build 5379, Leica, Wetzlar, Germany). For each scratch, at least three different positions were registered and, to measure the migration distances, for each position, at least 10 different cells were randomly selected. GraphPad Prism 5 software (GraphPad, San Diego, CA, USA) was used to perform the statistical analyses.

#### 4.2.7. Data Analysis

We reported data as mean ± standard error mean (SEM) of at least three independent experiments. Each experiment was conducted in triplicate. For the statistical analysis, we used the analysis of variance test. Bonferroni’s test was used to make multiple comparisons. We considered significant a *p*-value less than 0.05.

## 5. Conclusions

This study indicates that the three different peptide fractions from dehydrated potatoes possess anti-inflammatory and antioxidant activity and promote the cell migration in the IEC-6 monolayer. These findings highlight the nutraceutical potential of dehydrated potato peptides.

## Supplementary Materials

Supplementary materials can be found at https://www.mdpi.com/1422-0067/20/23/6087/s1. Tables S1, S2, and S3: Qualitative profile of peptides identified in the <1 kDa, 1–3 kDa, and 3–10 KDa fractions, respectively.

## Abbreviations

CVD	Cardiovascular disease
COX-2	Cycloxygenase-2
GI	Gastrointestinal
HO-1	Heme oxygenase-1
IBD	Inflammatory bowel disease
IECs	Intestinal epithelial cells
INF	Interferon-γ
iNOS	Inducible nitric oxide synthase
LPS	Lipopolysaccharide
mtROS	mitochondrial ROS
NMWL	Nominal molecular weight limit
ROS	Reactive oxygen species
SOD	Superoxide dismutase
TNF-α	Tumor necrosis factor-α
