# Supplementary material for: Anti-Inflammatory and Antioxidant Properties of Dehydrated Potato-Derived Bioactive Compounds in Intestinal Cells
Source: Int J Mol Sci. 2019 Dec 3;20(23):6087. doi: 10.3390/ijms20236087 (PMC6928682; doi:10.3390/ijms20236087)
Supplement: Supplementary file 1 [file ijms-20-06087-s001.pdf]

Table S1. Qualitative profile of peptides identified in the <1 kDa fraction.

n°	Peptide < 1 kDa	Mass	Length	Error (ppm)	m/z	Start	End	Protein
1.	D.ATPVL.D	499.3006	5	−1.4	500.3072	33	37	Kunitz-type inhibitor B
2.	Q.VGETL.L	517.2748	5	−0.1	518.2820	346	350	Patatin Group J−1
3.	N.GQGIF.F	520.2645	5	−0.8	521.2714	104	108	Kunitz-type inhibitor B
4.	F.TKSNL.A	561.3122	5	−0.6	562.3192	166	170	Patatin−2-Kuras 3
5.	K.FASIK.S	564.3271	5	−2.6	565.3329	242	246	Patatin−2-Kuras 3
6.	T.VGDPAL.L	570.3013	6	−2.4	571.3072	221	226	Patatin−2-Kuras 3
7.	D.VYLGK.S	578.3428	5	−1.9	579.3489	64	68	Kunitz-type inhibitor B
8.	D.IISTF.Y	579.3268	5	−2.0	580.3329	52	56	Kunitz-type inhibitor B
9.	T.VADPAL.L	584.3170	6	−2.3	585.3229	222	227	Patatin−01
10.	K.LLSDR.K	602.3387	5	0.6	302.1768	373	377	Patatin−01
11.	K.NGYPR.L	605.2921	5	0.0	303.6533	200	204	Cysteine protease inhibitor 1
12.	T.YEEAL.K	623.2802	5	−4.1	624.2849	362	366	Patatin−2-Kuras 3
13.	K.SLDYK.Q	624.3119	5	0.5	625.3195	248	252	Patatin Group J−1
14.	W.GPLRW.I	627.3492	5	−1.6	314.6814	279	283	Patatin−2-Kuras 3
15.	K.ELDPR.L	628.3180	5	−0.2	629.3251	43	47	Kunitz-type inhibitor B
16.	F.IGERY.V	636.3231	5	−0.8	319.1686	53	57	Putative cysteine proteinase inhibitor 1423
17.	M.YDGKY.F	644.2806	5	0.4	323.1477	125	129	Patatin−2-Kuras 3
18.	L.SVATRL.A	645.3810	6	−1.7	323.6972	231	236	Patatin Group J−1
19.	L.AKTPEL.D	657.3697	6	−2.8	329.6912	160	165	Probable Inactive Patatin−3-Kuras 1
20.	R.IISIGR.G	657.4174	6	−1.7	329.7154	52	57	Kunitz-type inhibitor B
21.	D.VYIKF.R	668.3897	5	−0.6	335.2019	124	128	Cysteine protease inhibitor 1
22.	A.TVGDPAL.L	671.3490	7	−1.2	672.3555	220	226	Patatin−2-Kuras 3
23.	E.FDKTY.T	672.3119	5	−0.7	337.1630	266	270	Patatin−2-Kuras 3
24.	F.WGALGGD.V	674.3024	7	−0.1	675.3096	57	63	Kunitz-type inhibitor B
25.	K.GIIPGII.P	681.4425	7	−0.2	682.4496	41	47	Patatin−11
26.	Y.IINNPL.L	682.4014	6	0.4	683.4089	63	68	Cysteine protease inhibitor 1
27.	I.PQFLGK.G	688.3907	6	−0.5	345.2025	89	94	Putative Kunitz-type tuber invertase inhibitor
28.	E.SSDDQF.N	697.2555	6	−2.6	698.2610	177	182	Kunitz-type inhibitor B
29.	Y.YLSTAF.Q	700.3431	6	0.1	701.3505	302	307	Patatin-T5
30.	M.TNAASSY.M	712.3028	7	2.6	713.3120	291	297	Patatin−2-Kuras 3
31.	Y.FEHGPK.I	713.3496	6	−1.0	357.6817	107	112	Patatin-T5
32.	Y.AISTSKL.K	718.4225	7	−0.1	360.2185	118	124	Kunitz-type inhibitor B
33.	F.HLVEPK.Y	721.4122	6	1.8	361.7141	108	113	Probable Inactive Patatin−3-Kuras 1
34.	Y.FEHGPH.I	722.3136	6	−0.5	362.1639	107	112	Patatin−2-Kuras 3
35.	T.IGVPTKL.Q	726.4639	7	−3.7	727.4685	51	57	Proteinase inhibitor 1
36.	L.AQVGENL.L	729.3657	7	0.8	730.3736	344	350	Patatin−01
37.	F.IGSSSHF.G	733.3395	7	−0.9	367.6767	97	103	Kunitz-type inhibitor B
38.	L.VQVGETL.L	744.4017	7	−0.8	745.4084	344	350	Patatin−13
39.	L.SIDGGGIK.G	745.3970	8	0.5	373.7060	33	40	Patatin−2-Kuras 3
40.	K.NIGGNFK.N	748.3868	7	1.5	749.3951	193	199	Cysteine protease inhibitor 1
41.	Y.GALGGDVY.T	750.3548	8	0.3	751.3623	58	65	Kunitz-type inhibitor B
42.	F.IPLSTNI.P	756.4381	7	−2.2	757.4437	101	107	Kunitz-type protease inhibitor
43.	L.VQVGENL.L	757.3970	7	0.3	758.4045	344	350	Patatin−05
44.	L.LVQVGEN.L	757.3970	7	1.0	758.4050	343	349	Patatin−05
45.	L.IFENQL.F	762.3912	6	−1.9	763.3970	107	112	Putative Kunitz-type tuber invertase inhibitor
46.	P.SGTPVRF.I	762.4024	7	1.7	763.4109	90	96	Kunitz-type inhibitor B
47.	K.LAQVDPK.F	769.4333	7	−2.2	385.7231	235	241	Patatin−2-Kuras 3
48.	L.LVQVGEK.L	771.4490	7	−0.3	772.4561	342	348	Patatin−2-Kuras 3
49.	R.AQEDPAF.A	776.3340	7	0.8	777.3419	237	243	Patatin−01
50.	F.NIPTVKL.C	783.4854	7	1.0	784.4935	118	124	Putative Kunitz-type tuber invertase inhibitor
51.	K.RIGERY.S	792.4242	6	−0.8	397.2191	57	62	Cysteine protease inhibitor 1
52.	K.IFEPSGF.H	795.3802	7	−1.0	398.6970	101	107	Probable Inactive Patatin−3-Kuras 1
53.	R.VHQALTE.V	796.4079	7	1.0	797.4160	144	150	Patatin−01
54.	K.GIIPATIL.E	796.5058	8	−1.0	399.2598	41	48	Patatin−2-Kuras 3
55.	F.NLVDGDVA.A	801.3868	8	−3.3	802.3915	201	208	Probable Inactive Patatin−3-Kuras 1
56.	L.AQVDPKF.A	803.4177	7	−0.3	402.7160	236	242	Patatin−2-Kuras 3
57.	Y.FVTHTSN.G	804.3766	7	1.5	403.1962	199	205	Patatin−2-Kuras 3
58.	W.ILAIQQM.T	815.4575	7	−1.9	816.4633	284	290	Patatin−2-Kuras 3
59.	K.TNKPVIF.T	817.4698	7	−2.3	409.7412	160	166	Patatin−01
60.	E.LVLPEVY.L	831.4742	7	0.1	832.4816	43	49	Cysteine protease inhibitor 1
61.	W.MLAIQQM.T	833.4139	7	−1.4	834.4200	285	291	Patatin Group J−1
62.	E.FLEGQLQ.K	833.4283	7	−0.7	834.4349	50	56	Patatin−01
63.	V.FDNILGY.A	840.4017	7	−1.8	841.4075	93	99	Proteinase inhibitor 1
64.	A.VATVGDPAL.L	841.4545	9	−0.4	842.4615	218	226	Patatin−2-Kuras 3

65.	L.LAQVGENL.L	842.4498	8	−1.4	843.4559	343	350	Patatin−01
66.	T.KVNDEQL.I	844.4290	7	−0.5	423.2216	143	149	Cysteine protease inhibitor 1
67.	G.SPLPKPVL.Y	849.5323	8	−1.2	425.7729	34	41	Kunitz-type protease inhibitor
68.	L.TEVAISSF.D	852.4229	8	0.1	853.4302	148	155	Patatin−2-Kuras 3
69.	L.LVQVGETL.L	857.4858	8	−0.3	858.4929	343	350	Patatin Group J−1
70.	Q.HMSIPQF.L	858.4058	7	−1.5	859.4118	91	97	Cysteine protease inhibitor 1
71.	F.NLVDGAVAT.V	858.4447	9	−0.9	859.4512	212	220	Patatin−2-Kuras 3
72.	F.YFDHGPK.I	862.3973	7	0.3	432.2061	94	100	Probable Inactive Patatin−3-Kuras 1
73.	D.YYLSTAF.Q	863.4065	7	0.0	864.4138	301	307	Patatin-T5
74.	Y.GALGGDVYL.G	863.4388	9	0.8	864.4468	58	66	Kunitz-type inhibitor B
75.	L.LVQVGENL.L	870.4811	8	−0.5	871.4879	343	350	Patatin−05
76.	R.HSQNNYL.R	874.3933	7	−2.1	875.3987	311	317	Patatin−2-Kuras 3
77.	L.DVTGKELD.S	875.4236	8	0.1	876.4310	38	45	Kunitz-type inhibitor B
78.	F.YFQHGPH.I	884.3929	7	−0.6	443.2035	106	112	Patatin−11
79.	L.LVQVGEKL.L	884.5331	8	−1.0	885.5395	342	349	Patatin−2-Kuras 3
80.	F.YFEHGPH.I	885.3770	7	−0.3	443.6956	106	112	Patatin−01
81.	R.LAQEDPAF.S	889.4181	8	−1.6	890.4240	236	243	Patatin Group J−1
82.	F.IPLSTNIF.K	903.5065	8	0.7	904.5144	101	108	Kunitz-type protease inhibitor
83.	Y.TAEEAAKW.G	904.4290	8	−3.5	453.2202	271	278	Patatin−2-Kuras 3
84.	E.NALTGTTTK.A	905.4818	9	−1.6	906.4876	323	331	Patatin−01
85.	T.ISNVHILL.T	907.5491	8	−0.6	454.7815	69	76	Proteinase inhibitor 1
86.	F.FGPKYDGK.Y	910.4548	8	−1.2	456.2341	122	129	Patatin−01
87.	N.LPSDATPVL.D	911.4963	9	1.1	912.5046	29	37	Kunitz-type inhibitor B
88.	G.AVATVGDPAL.L	912.4916	10	1.0	913.4998	217	226	Patatin−2-Kuras 3
89.	K.VGVVIQNGK.R	912.5392	9	−1.0	457.2765	194	202	Kunitz-type protease inhibitor
90.	K.LAQVDPKF.A	916.5018	8	−3.3	459.2567	235	242	Patatin−2-Kuras 3
91.	F.LGEGTPVVF.V	917.4858	9	0.6	918.4936	98	106	Cysteine protease inhibitor 1
92.	K.RIISTFW.F	921.5072	7	−2.2	922.5125	51	57	Kunitz-type inhibitor B
93.	K.ELNPDSSY.R	923.3872	8	−3.5	924.3913	47	54	Putative Kunitz-type tuber invertase inhibitor
94.	F.GPKYDGKY.L	926.4497	8	−1.6	464.2314	123	130	Patatin−01
95.	F.GPMYDGKY.F	929.3953	8	−3.1	465.7035	122	129	Patatin−2-Kuras 3
96.	R.RAQEDPAF.A	932.4352	8	−1.4	467.2242	236	243	Patatin−01
97.	F.WGALGGDVY.L	936.4341	9	−1.4	937.4400	57	65	Kunitz-type inhibitor B
98.	K.VGVVHQNGK.R	936.5141	9	−1.4	469.2628	194	202	Kunitz-type inhibitor B
99.	F.AAAKDIIPF.Y	944.5331	9	−0.4	473.2736	97	105	Patatin−2-Kuras 2
100.	A.AKDIVPFY.F	951.5065	8	−2.1	952.5118	99	106	Patatin−2-Kuras 3
101.	L.AKSPELDAK.M	957.5131	9	−1.3	320.1779	172	180	Patatin−01
102.	T.VLSIDGGGIK.G	957.5494	10	−1.2	479.7814	31	40	Patatin−01
103.	E.FLEGQLQK.M	961.5233	8	−3.1	481.7674	50	57	Patatin−01
104.	W.KVGDYDASL.G	966.4658	9	−1.4	484.2386	132	140	Kunitz-type inhibitor B
105.	Y.LLQVLQEK.L	969.5859	8	−0.3	485.8001	130	137	Patatin−15
106.	D.VIGGTSTGGLL.T	973.5444	11	0.7	974.5523	72	82	Patatin−2-Kuras 3
107.	F.DVIGGTSTGGL.L	975.4873	11	−3.2	976.4915	71	81	Patatin−01
108.	L.LSLGTGTNSE.F	977.4666	10	0.1	489.7406	257	266	Patatin−05
109.	G.ESPLPKPVL.Y	978.5750	9	−0.1	979.5822	33	41	Kunitz-type protease inhibitor
110.	Y.FEHGPHIF.N	982.4661	8	−3.4	983.4700	107	114	Patatin−01
111.	K.IFQSSGSIF.G	984.4916	9	−3.1	985.4959	113	121	Patatin-T5
112.	Y.LMQVLQEK.L	987.5423	8	−3.7	494.7766	131	138	Patatin−01
113.	N.FKNGYPRL.V	993.5396	8	−0.5	497.7768	198	205	Cysteine protease inhibitor 1
114.	N.IIKNPLLGAG.K	994.6175	10	−4.0	498.3141	57	66	Putative Kunitz-type tuber invertase inhibitor
115.	E.NALTGTATTF.D	995.4924	10	−3.6	996.4961	310	319	Probable Inactive Patatin−3-Kuras 1
116.	Y.FLQVLQEK.L	1003.5702	8	−0.5	502.7921	130	137	Patatin−2-Kuras 3
117.	L.VNENPLDVL.E	1011.5237	9	−2.1	1.012.5288	208	216	Kunitz-type protease inhibitor
118.	D.VGPSGTPVRF.I	1015.5450	10	−1.4	508.7791	87	96	Kunitz-type inhibitor B
119.	K.MDNNADARL.A	1018.4502	9	−5.2	510.2297	58	66	Patatin−01
120.	A.PTYFPPHY.F	1020.4705	8	−3.0	511.2410	191	198	Patatin−2-Kuras 3
121.	M.IGSSSHFGPH.I	1024.4727	10	−3.5	513.2418	97	106	Kunitz-type inhibitor B
122.	D.ILLNGSPVTL.W	1025.6121	10	1.0	1.026.6204	74	83	Proteinase inhibitor 1
123.	A.PIYFPPHY.F	1032.5068	8	−2.5	517.2594	179	186	Patatin−2-Kuras 1
124.	F.IPLSTNIFE.D	1032.5491	9	1.6	1.033.5580	101	109	Kunitz-type protease inhibitor
125.	E.NALTGTTTEM.D	1037.4700	10	−3.8	1038.4733	322	331	Patatin−2-Kuras 3
126.	L.IIKNPLLGGGA.L	1051.6389	11	−3.7	526.8248	61	71	Kunitz-type inhibitor c
127.	F.DVIGGTGTGGLL.T	1058.5608	12	−2.4	1059.5656	59	70	Probable Inactive Patatin−3-Kuras 1
128.	S.KVGVVHQNGK.R	1064.6090	10	−2.7	533.3104	193	202	Kunitz-type inhibitor B
129.	T.SESPLPKPVL.Y	1065.6069	10	−3.1	533.8091	32	41	Kunitz-type protease inhibitor
130.	Y.IIKNPLLGAGA.V	1065.6545	11	−1.2	533.8339	57	67	Putative Kunitz-type tuber invertase inhibitor
131.	V.VGVVIQNGKR.V	1068.6404	10	−1.6	535.3266	194	203	Kunitz-type protease inhibitor
132.	K.GIIPATILEF.L	1072.6168	10	−1.7	1073.6223	41	50	Patatin−2-Kuras 3

133.	F.FGPKYDGKY.L	1073.5182	9	−1.3	537.7657	122	130	Patatin−01
134.	E.FDKTHTAEE.T	1076.4774	9	−0.4	539.2458	267	275	Patatin−01
135.	T.FWGALGGDVY.L	1083.5026	10	−3.3	1.084.5062	56	65	Kunitz-type inhibitor B
136.	F.DVIGGTSTGGLL.T	1088.5713	12	0.0	1089.5786	71	82	Patatin−2-Kuras 3
137.	K.VGVVHQNGKR.R	1092.6152	10	−3.9	547.3127	194	203	Kunitz-type inhibitor B
138.	F.AAAKDIVPFY.F	1093.5807	10	0.7	1094.5887	97	106	Patatin−2-Kuras 3
139.	L.LSLGTGTTSEF.D	1111.5397	11	−2.4	1112.5443	257	267	Patatin−01
140.	F.QDLHSQNNY.L	1117.4789	9	1.7	559.7477	309	317	Patatin−01
141.	Y.FDVIGGTSTGGL.L	1122.5557	12	0.9	1123.5640	70	81	Patatin−2-Kuras 3
142.	L.LSLGTGTNSEF.D	1124.5349	11	−0.7	563.2744	257	267	Patatin−05
143.	L.VKDNPLDVSF.M	1132.5764	10	1.2	567.2961	208	217	Kunitz-type inhibitor B
144.	A.TRLAQEDPAF.S	1146.5669	10	−0.4	574.2905	234	243	Patatin Group J−1
145.	A.PIYFPPHHF.V	1153.5708	9	−0.7	385.5306	192	200	Patatin Group J−1
146.	L.VNENPLDVLF.T	1158.5920	10	−1.4	580.3025	208	217	Kunitz-type protease inhibitor
147.	N.IIKNPLLGAGAV.P	1164.7230	12	−1.4	583.3679	57	68	Putative Kunitz-type tuber invertase inhibitor
148.	F.DIKTNKPVIF.T	1173.6758	10	−1.4	587.8444	157	166	Patatin−01
149.	F.DVIGGTSTGGLLT.A	1189.6190	13	0.1	1190.6265	71	83	Patatin−01
150.	Y.FDVIGGTGTGGLL.T	1205.6292	13	−3.2	1206.6326	58	70	Probable Inactive Patatin−3-Kuras 1
151.	R.YIINNPLLGAGA.V	1214.6659	12	−1.6	1.215.6705	62	73	Cysteine protease inhibitor 1
152.	S.KVGVVHQNGKR.R	1220.7102	11	−0.9	407.9103	193	203	Kunitz-type inhibitor B
153.	K.LLFPGMPKPLN.Y	1225.6892	11	−1.0	613.8513	84	94	Putative Kunitz-type tuber invertase inhibitor
154.	F.IGSSSHFGQGIF.E	1235.5935	12	0.4	618.8043	97	108	Kunitz-type inhibitor B
155.	Y.FDVIGGTSTGGLL.T	1235.6398	13	−0.3	618.8270	70	82	Patatin−01
156.	Y.FATNTINGDKY.E	1242.5880	11	−1.0	622.3007	200	210	Patatin−01
157.	G.PEVYDQDGNPL.I	1245.5513	11	−2.7	623.7812	44	54	Kunitz-type inhibitor C
158.	A.LVKDNPLDVSF.M	1245.6604	11	−1.1	623.8368	207	217	Kunitz-type inhibitor B
159.	Y.FVTHTSNGDKY.E	1267.5833	11	0.1	634.7990	199	209	Patatin−2-Kuras 3
160.	C.PEVYDQDGHPL.Q	1268.5673	11	−0.9	635.2903	44	54	Kunitz-type trypsin inhibitor
161.	L.DTNGKELNPDSS.Y	1275.5579	12	−0.8	638.7857	42	53	Putative Kunitz-type tuber invertase inhibitor
162.	L.AIQQMTNAASSY.M	1283.5815	12	−2.5	642.7964	286	297	Patatin−2-Kuras 3
163.	K.SVSEDNHETYE.V	1308.5106	11	−0.6	655.2622	341	351	Probable Inactive Patatin−3-Kuras 1
164.	R.LALVNENPLDVL.F	1308.7289	12	0.2	655.3718	205	216	Putative Kunitz-type tuber invertase inhibitor
165.	G.IINNPLIGAGAVY.G	1313.7343	13	−0.8	657.8739	61	73	Kunitz-type trypsin inhibitor
166.	L.IIKNPLLGGGAVY.V	1313.7706	13	0.6	657.8930	61	73	Kunitz-type inhibitor C
167.	L.LKKPVSKDSPET.Y	1327.7346	12	−1.1	443.5850	350	361	Patatin−2-Kuras 3
168.	Y.IIKNPLLGAGAVY.L	1327.7864	13	0.6	664.8993	57	69	Putative Kunitz-type tuber invertase inhibitor
169.	Y.NSDVGPSGTPVRF.I	1331.6470	13	−0.6	666.8304	84	96	Kunitz-type inhibitor B
170.	Y.FDVIGGTSTGGLLT.A	1336.6874	14	0.2	669.3511	70	83	Patatin−01
171.	R.YNSDVGPSGTPVR.F	1347.6418	13	−1.5	674.8272	83	95	Kunitz-type inhibitor B
172.	L.AAVDDDKDFIPF.V	1351.6295	12	3.1	676.8241	200	211	Putative cysteine proteinase inhibitor 1423
173.	F.IGSSSHFGQGIFE.N	1364.6360	13	1.2	683.3261	97	109	Kunitz-type inhibitor B
174.	D.YFDVIGGTGTGGLL.T	1368.6925	14	−1.8	685.3523	57	70	Probable Inactive Patatin−3-Kuras 1
175.	V.FQDLHSQNNYL.R	1377.6313	11	0.2	689.8231	308	318	Patatin−01
176.	S.LETGGTIGQADSSY.N	1397.6310	14	0.2	699.8229	145	158	Kunitz-type protease inhibitor
177.	D.YFDVIGGTSTGGLL.T	1398.7031	14	−2.5	700.3571	69	82	Patatin−01
178.	R.VHQALTEVAISSF.D	1400.7300	13	1.4	701.3732	144	156	Patatin−01
179.	G.PEVYDQDGNPLR.F	1401.6525	12	−0.4	701.8333	44	55	Kunitz-type inhibitor C
180.	L.VTVDDDKDFIPF.V	1409.6714	12	−2.3	705.8414	206	217	Cysteine protease inhibitor 1
181.	L.LETGGTIGQADSSW.F	1420.6470	14	0.9	711.3314	145	158	Kunitz-type inhibitor B
182.	Y.IINNPLLGAGAVYL.Y	1426.8184	14	0.6	714.4169	63	76	Cysteine protease inhibitor 1
183.	Y.IIKNPLLGGGAVYL.D	1426.8547	14	1.1	714.4354	61	74	Kunitz-type inhibitor C
184.	R.LALVKDNPLDVSF.K	1429.7816	13	−1.9	715.8967	205	217	Kunitz-type inhibitor B
185.	A.MITTPNENNRPF.A	1432.6769	12	0.5	717.3461	85	96	Patatin−01
186.	N.VVTGGNVGNENDIF.V	1433.6787	14	0.2	717.8467	148	161	Kunitz-type inhibitor C
187.	L.DTNGKELNPDSSY.R	1438.6212	13	1.5	720.3190	42	54	Putative Kunitz-type tuber invertase inhibitor
188.	M.VVTGGKVGNENDIF.N	1447.7307	14	−3.2	724.8703	150	163	Cysteine protease inhibitor 1
189.	R.LALVNENPLDVLF.Q	1455.7972	13	2.7	728.9078	205	217	Putative Kunitz-type tuber invertase inhibitor
190.	R.RLALVNENPLDVL.F	1464.8300	13	0.3	733.4225	204	216	Putative Kunitz-type tuber invertase inhibitor
191.	T.VFQDLHSQNNYL.R	1476.6997	12	1.0	739.3578	307	318	Patatin−01
192.	R.YIINNPLLGAGAVY.L	1476.7976	14	0.8	739.4045	62	75	Cysteine protease inhibitor 1
193.	E.NALTGTTTKADDASE.A	1493.6846	15	−3.0	747.8474	323	337	Patatin−01
194.	R.YNSDVGPSGTPVRF.I	1494.7102	14	0.1	748.3630	83	96	Kunitz-type inhibitor B
195.	Y.FATNTINGDKYEF.N	1518.6990	13	−0.1	760.3567	200	212	Patatin−01
196.	L.SIDGGGIKGIIPATIL.E	1523.8922	16	1.2	762.9543	33	48	Patatin−2-Kuras 3

197.	Y.FVTHTSNGDKYEF.N	1543.6943	13	0.1	772.8545	199	211	Patatin−2-Kuras 3
198.	W.MLVIQQMTEAASSY.M	1570.7371	14	−3.2	786.3733	285	298	Patatin−01
199.	G.LVLPEVYDQDGNPL.I	1570.7878	14	−2.0	786.3996	41	54	Kunitz-type inhibitor C
200.	L.TAMITTPNENNRPF.A	1604.7617	14	0.0	803.3881	83	96	Patatin−01
201.	L.LSLGTGTNSEFDKTY.T	1631.7678	15	1.6	816.8925	256	270	Patatin
202.	M.RFNSDVGPSGTPVRF.V	1634.8164	15	1.0	818.4163	82	96	Kunitz-type inhibitor B
203.	F.RYNSDVGPSGTPVRF.I	1650.8114	15	0.2	826.4131	82	96	Kunitz-type inhibitor B
204.	Y.EFNLVDGAVATVGDPAL.L	1686.8464	17	1.8	844.4320	210	226	Patatin−2-Kuras 3
205.	R.LADYFDVIGGTSTGGLL.T	1697.8512	17	1.1	849.9338	66	82	Patatin−01

Table S2. **Peptides identified in the 1–3 kDa fraction.**

n°	Peptide (1–3 kDa)	Mass	Length	Error (ppm)	m/z	Start	End	Protein
1.	F.TKSNL.A	561.3122	5	−3.2	562.3177	166	170	Patatin−2-Kuras 3
2.	T.YEEAL.K	623.2802	5	−0.9	624.2869	362	366	Patatin−2-Kuras 3
3.	R.LADYF.D	627.2904	5	−1.2	628.2969	66	70	Patatin−2-Kuras 3
4.	L.AKSPEL.D	643.3541	6	−2.6	322.6835	171	176	Patatin−2-Kuras 3
5.	L.NGSPVTL.D	686.3599	7	2.8	687.3669	41	47	Serine protease inhibitor
6.	F.AISTSKL.C	718.4225	7	2.7	719.4294	91	97	Kunitz-type proteinase inhibitor
7.	Y.FQHGPH.I	721.3296	6	−2.2	361.6713	107	112	Patatin−05
8.	L.IGVPTKL.A	726.4639	7	0.1	727.4690	15	21	Serine protease inhibitor
9.	L.AQVGENL.L	729.3657	7	−3.8	730.3702	344	350	Patatin−01
10.	F.IGSSSHF.G	733.3395	7	3.4	734.3469	97	103	Kunitz-type inhibitor B
11.	F.SISTSKL.C	734.4174	7	0.8	368.2151	118	124	Kunitz-type inhibitor B
12.	Y.KQMLLL.S	744.4568	6	−1.3	373.2352	251	256	Patatin−2-Kuras 3
13.	L.VQVGENL.L	757.3970	7	0.1	758.4043	343	349	Patatin−07
14.	L.DVTGKEL.D	760.3967	7	0.9	381.2047	38	44	Serine protease inhibitor 5
15.	R.HSQNNY.L	761.3093	6	0.4	762.3168	311	316	Patatin−2-Kuras 3
16.	H.TSNGARY.E	767.3562	7	−3.1	384.6842	203	209	Patatin-B2
17.	K.LAQVDPK.F	769.4333	7	−3.4	385.7226	235	241	Patatin−2-Kuras 3
18.	L.VQVGEKL.L	771.4490	7	−1.8	386.7311	343	349	Patatin−2-Kuras 3
19.	R.AQEDPAF.A	776.3340	7	−4.3	777.3380	237	243	Patatin−01
20.	K.GIIPATIL.E	796.5058	8	−2.6	399.2592	41	48	Patatin−2-Kuras 3
21.	F.NLVDGDVA.A	801.3868	8	−4.7	802.3904	201	208	Probable Inactive Patatin−3-Kuras 1
22.	L.AQVDPKF.A	803.4177	7	−2.7	402.7151	236	242	Patatin−2-Kuras 3
23.	L.SLSVATKL.A	817.4909	8	−1.5	409.7521	228	235	Patatin−2-Kuras 3
24.	L.RVQENAL.T	828.4453	7	−2.6	415.2289	318	324	Patatin−06
25.	L.LSVSVATR.R	831.4814	8	−2.1	416.7471	228	235	Patatin−01
26.	L.EFLEGQL.Q	834.4123	7	−1.6	835.4183	49	55	Patatin−2-Kuras 3
27.	A.VATVGDPAL.L	841.4545	9	−1.5	842.4606	218	226	Patatin−2-Kuras 3
28.	L.LAQVGENL.L	842.4498	8	−1.8	422.2314	343	350	Patatin−01
29.	W.KVNDEEL.V	845.4130	7	2.2	423.7134	138	144	Putative cysteine proteinase inhibitor 1423
30.	F.KNGYPRL.A	846.4711	7	2.3	424.2425	193	199	Putative cysteine proteinase inhibitor 1423
31.	L.TEVAISSF.D	852.4229	8	−0.4	853.4298	148	155	Patatin−2-Kuras 3
32.	L.LVQVGETL.L	857.4858	8	−0.4	858.4927	330	337	Patatin−2-Kuras 1
33.	R.HSQNNYL.R	874.3933	7	−0.9	875.3998	311	317	Patatin−15
34.	A.NEIVPFY.F	880.4330	7	−3.1	441.2224	100	106	Patatin−01
35.	A.KDIVPFY.F	880.4694	7	−3.3	441.2405	100	106	Patatin−2-Kuras 3
36.	L.LVQVGEKL.L	884.5331	8	−3.0	443.2725	342	349	Patatin−2-Kuras 3
37.	F.YFEHGPH.I	885.3770	7	−4.1	443.6939	106	112	Patatin−2-Kuras 3
38.	Y.TAEEAAKW.G	904.4290	8	−2.9	453.2205	271	278	Patatin−2-Kuras 3
39.	K.MDNNADAR.L	905.3661	8	−4.5	453.6883	58	65	Patatin−01
40.	L.ISNVHILL.N	907.5491	8	−0.5	454.7802	33	40	Serine protease inhibitor
41.	F.FGPKYDGK.Y	910.4548	8	−3.1	456.2333	122	129	Patatin−01
42.	K.VGVVIQNGK.R	912.5392	9	1.2	457.2758	143	151	Aspartic protease inhibitor 3
43.	K.LAQVDPKF.A	916.5018	8	−2.4	459.2571	235	242	Patatin−2-Kuras 3
44.	F.LGKGTPVVF.V	916.5381	9	0.2	459.2750	93	101	Putative cysteine proteinase inhibitor 1423
45.	Y.RIISTFW.G	921.5072	7	1.0	461.7599	51	57	Kunitz-type inhibitor B
46.	K.GIIPATILE.F	925.5484	9	−1.3	463.7809	41	49	Patatin−2-Kuras 3
47.	F.GPMYDGKY.F	929.3953	8	−2.4	465.7038	122	129	Patatin−2-Kuras 3
48.	R.RAQEDPAF.A	932.4352	8	−3.2	467.2234	236	243	Patatin−01
49.	F.WGALGGDVY.L	936.4341	9	5.7	937.4437	57	65	Kunitz-type inhibitor B

50.	K.VGVVHQNGK.R	936.5141	9	−0.7	469.2623	194	202	Kunitz-type inhibitor B
51.	F.AAAKDIIPF.Y	944.5331	9	−2.0	473.2729	97	105	Patatin−2-Kuras 2
52.	W.KVGDYDASL.G	966.4658	9	−1.4	484.2380	132	140	Kunitz-type inhibitor B
53.	E.SPLPKPVL.D	978.5750	9	1.5	490.2939	1	9	Aspartic protease inhibitor 11
54.	L.QHMSIPQF.L	986.4644	8	2.0	494.2389	85	92	Putative cysteine proteinase inhibitor 1423
55.	Y.SIVGPTHSPL.R	1006.5447	10	−0.5	504.2778	101	110	Cysteine protease inhibitor 3
56.	L.VNENPLDVL.F	1011.5237	9	0.8	506.7679	179	187	Kunitz-type proteinase inhibitor group A1
57.	A.PIYFPPHY.F	1032.5068	8	−1.7	517.2598	179	186	Patatin−2-Kuras 1
58.	E.NALTGTTTEM.D	1037.4700	10	−3.6	519.7404	322	331	Patatin−2-Kuras 3
59.	F.DVIGGTGTGGLL.T	1058.5608	12	−3.6	1059.5642	59	70	Probable Inactive Patatin−3-Kuras 1
60.	D.IKTNKPVIF.T	1058.6488	9	−4.8	530.3292	157	165	Patatin−2-Kuras 3
61.	A.KLEEMVTVL.S	1060.5839	9	−3.6	531.2973	24	32	Patatin-B2
62.	L.ALNNKPYPF.G	1062.5498	9	−0.9	532.2800	177	185	Cysteine protease inhibitor 3
63.	K.VGVVIQNGKR.R	1068.6404	10	0.9	535.3260	143	152	Aspartic protease inhibitor 3
64.	F.FGPKYDGKY.L	1073.5182	9	−3.2	537.7646	122	130	Patatin−01
65.	N.TINGDKYEF.N	1085.5029	9	−1.4	543.7580	204	212	Patatin−01
66.	F.DVIGGTSTGGLL.T	1088.5713	12	5.3	1089.5809	59	70	Kunitz-type proteinase inhibitor
67.	L.EFLEGQLQK.M	1090.5658	9	−0.5	546.2899	49	57	Patatin−01
68.	K.VGVVHQNGKR.R	1092.6152	10	−1.1	547.3129	194	203	Kunitz-type inhibitor B
69.	F.VTHTSNGARY.E	1104.5312	10	−3.2	369.1832	200	209	Patatin-B2
70.	F.AAAKDIIPFY.F	1107.5964	10	−1.1	554.8049	97	106	Patatin−2-Kuras 2
71.	L.LSLGTGTTSEF.D	1111.5397	11	−0.9	556.7766	257	267	Patatin−01
72.	F.QDLHSQNNY.L	1117.4789	9	−1.4	559.7459	309	317	Patatin−01
73.	Y.FDVIGGTSTGGL.L	1122.5557	12	−5.1	1123.5573	70	81	Patatin−2-Kuras 3
74.	L.LSLGTGTNSEF.A	1124.5349	11	−0.7	563.2744	244	254	Probable Inactive Patatin−3-Kuras 1
75.	L.VKDNPLDVSF.K	1132.5764	10	0.8	567.2942	200	209	Serine protease inhibitor 5
76.	A.TKLAQVDPKF.A	1145.6444	10	−1.5	382.8882	233	242	Patatin−2-Kuras 3
77.	A.TRLAQEDPAF.S	1146.5669	10	−0.1	574.2906	221	230	Patatin−2-Kuras 1
78.	L.VKDNPLDISF.N	1146.5920	10	1.7	574.3025	181	190	Kunitz-type proteinase inhibitor
79.	A.PIYFPPHHF.V	1153.5708	9	−2.4	385.5300	191	199	Patatin-B2
80.	F.KLVVQVTPSM.G	1157.6478	11	1.3	579.8302	107	117	Proteinase inhibitor I
81.	L.VTVDDDKDFL.P	1165.5503	10	1.8	583.7817	204	213	Kunitz-type trypsin inhibitor
82.	L.QEVDNNKDAR.L	1187.5531	10	−3.4	396.8570	56	65	Patatin−2-Kuras 3
83.	L.TGTTTKADDASE.A	1195.5204	12	−5.4	598.7642	326	337	Patatin−01
84.	R.AQEDPAFASIR.S	1203.5884	11	−2.3	602.8001	237	247	Patatin−01
85.	Y.FDVIGGTGTGGLL.T	1205.6292	13	−4.1	603.8194	58	70	Probable Inactive Patatin−3-Kuras 1
86.	A.IQQMTNAASSY.M	1212.5444	11	−1.0	607.2789	287	297	Patatin−2-Kuras 3
87.	E.MDDASEANMEL.L	1224.4639	11	−3.0	1225.4675	331	341	Patatin−2-Kuras 3
88.	L.DVTGKELDSRL.S	1231.6407	11	−0.4	616.8255	38	48	Kunitz-type inhibitor B
89.	F.IGSSSHFGQGIF.E	1235.5935	12	0.2	618.8022	97	108	Kunitz-type inhibitor B
90.	Y.FDVIGGTSTGGLL.T	1235.6398	13	−1.8	618.8260	70	82	Patatin−01
91.	L.PEVYDQDGNPL.R	1245.5513	11	2.9	623.7828	4	14	20 kDa kunitz-type proteinase inhibitor
92.	Y.FVTHTSNGDKY.E	1267.5833	11	−3.3	634.7968	199	209	Patatin−2-Kuras 3
93.	L.PEVYDQDGHPL.R	1268.5673	11	−0.5	635.2886	44	54	Kunitz-type trypsin inhibitor
94.	L.QKMDNNADARL.A	1274.6038	11	−2.3	425.8742	56	66	Patatin−01
95.	L.AIQQMTNAASSY.M	1283.5815	12	−0.5	642.7977	286	297	Patatin−2-Kuras 3
96.	R.LAQEDPAFASIK.S	1288.6663	12	−2.5	645.3388	223	234	Probable Inactive Patatin−3-Kuras 1
97.	M.ITTPNENNRPF.A	1301.6364	11	−2.2	434.8851	86	96	Patatin−2-Kuras 3
98.	K.SVSEDNHETYE.V	1308.5106	11	−1.7	655.2615	341	351	Probable Inactive Patatin−3-Kuras 1
99.	Y.IINNPLLGAGAVY.L	1313.7343	13	1.8	657.8735	63	75	Putative Kunitz-type invertase inhibitor
100.	L.LKKPVSKDSPET.Y	1327.7346	12	−2.5	664.8729	350	361	Patatin−2-Kuras 3
101.	Y.IIKNPLLGAGAVY.L	1327.7864	13	−1.3	664.8975	57	69	Putative Kunitz-type tuber invertase inhibitor
102.	Y.NSDVGPSGTPVRF.I	1331.6470	13	−0.6	666.8282	84	96	Kunitz-type inhibitor B
103.	Y.FDVIGGTSTGGLLT.S	1336.6874	14	−1.6	669.3499	70	83	Patatin Group D−2
104.	M.ILLVQVGENLLK.K	1337.8282	12	−1.5	669.9204	328	339	Probable Inactive Patatin−3-Kuras 1
105.	R.YNSDVGPSGTPVR.F	1347.6418	13	3.4	674.8280	83	95	Kunitz-type inhibitor B
106.	L.AAVDDDKDFIPF.V	1351.6295	12	0.6	676.8203	200	211	Putative cysteine proteinase inhibitor 1423
107.	T.VFQDLHSQNNY.L	1363.6157	11	−0.9	682.8145	307	317	Patatin−01
108.	M.ITTPNENNRPFA.A	1372.6735	12	−0.8	687.3434	86	97	Patatin−01
109.	L.LETGGTIGQADSSY.F	1397.6310	14	3.3	699.8229	116	129	Kunitz-type proteinase inhibitor group A1
110.	R.VHQALTEVAISSF.D	1400.7300	13	−1.6	701.3712	144	156	Patatin−01
111.	F.NLVDGAVATVGDPAL.L	1410.7355	15	2.9	706.3749	200	214	Kunitz-type proteinase inhibitor
112.	F.DKTHTAQETAKW.G	1414.6841	12	−2.8	472.5673	267	278	Patatin−07
113.	L.RVQENALTGTTTE.M	1418.7001	13	−3.2	710.3550	306	318	Patatin−2-Kuras 4
114.	L.LETGGTIGQADSSW.F	1420.6470	14	2.9	711.3306	145	158	Kunitz-type inhibitor B
115.	Y.STAAAPTYFPPHY.F	1421.6615	13	−0.2	711.8379	187	199	Patatin−08

116.	Y.IIKNPLLGGGAVYL.Y	1426.8547	14	2.2	714.4340	58	71	Putative cysteine proteinase inhibitor 1423
117.	A.MITTPNENNRPF.A	1432.6769	12	−2.9	717.3436	85	96	Patatin−01
118.	L.VVTGGNVGNENDIF.K	1433.6787	14	2.7	717.8463	145	158	Putative cysteine proteinase inhibitor 1423
119.	R.LALVKDNPLDIS.F	1443.7972	13	0.1	722.9033	205	217	Kunitz-type inhibitor B
120.	T.VFQDLHSQNNYL.R	1476.6997	12	−4.2	739.3541	307	318	Patatin−01
121.	E.NALTGTTTKADDASE.A	1493.6846	15	−1.2	747.8487	323	337	Patatin−01
122.	T.AMITTPNENNRPF.A	1503.7140	13	−0.7	752.8637	84	96	Patatin−01
123.	Y.FATNTINGDKYEF.N	1518.6990	13	−2.1	760.3552	200	212	Patatin−02
124.	L.SIDGGGIKGIIPATIL.E	1523.8922	16	−2.1	762.9518	33	48	Patatin−2-Kuras 3
125.	E.FNLVDGGVATVGDPAL.L	1543.7882	16	−0.1	772.9013	199	214	Patatin−2-Kuras 4
126.	L.LETGGTIGQADSSYF.K	1544.6995	15	1.7	773.3560	116	130	Kunitz-type proteinase inhibitor group A1
127.	E.NALTGTTTKADDASEA.N	1564.7217	16	−4.5	783.3646	323	338	Patatin−02
128.	R.LFDNILGSVVQIPR.V	1569.8878	14	1.2	785.9468	92	105	Protease inhibitor I
129.	W.MLVIQQMTEAASSY.M	1570.7371	14	−3.4	786.3731	285	298	Patatin−01
130.	L.VLPEVYDQDGNPL.R	1570.7878	14	3.9	786.4019	1	14	20 kDa kunitz-type proteinase inhibitor
131.	L.ISNVHILLNGSPVTL.D	1575.8984	15	3.7	788.9570	33	47	Serine protease inhibitor
132.	L.TAMITTPNENNRPF.A	1604.7617	14	−1.2	803.3872	83	96	Patatin−01
133.	L.LKKPVSKDSPETYE.E	1619.8406	14	−2.4	540.9528	350	363	Patatin−2-Kuras 3
134.	L.LSLGTGTNSEFDKTY.T	1631.7678	15	−1.1	816.8903	256	270	Patatin−2-Kuras 3
135.	F.RFNSDVGPSGTPVRF.I	1634.8164	1.5	1.5	545.9453	82	96	Serine protease inhibitor 5
136.	F.RYNSDVGPSGTPVRF.I	1650.8114	15	−0.2	826.4103	82	96	Kunitz-type inhibitor B
137.	Y.EFNLVDGAVATVGDPAL.L	1686.8464	17	−2.6	844.4283	210	226	Patatin−2-Kuras 3
138.	F.LEGQLQEVDNNKDAR.L	1727.8438	15	−1.0	576.9546	51	65	Patatin−2-Kuras 3
139.	Y.EFNLVDGAVATVGDPALL.S	1799.9304	18	−0.5	900.9720	210	227	Patatin−2-Kuras 3
140.	F.LEGQLQEVDNNKDARL.A	1840.9279	16	−1.4	614.6490	51	66	Patatin−2-Kuras 3

Table S3. List of peptides identified in the 3–10 KDa fraction

n°	Peptide (3–10 kDa)	Mass	Length	Error (ppm)	m/z	Start	End	Protein
206.	F.TKSNL.A	561.3122	5	−3.1	562.3177	166	170	Patatin−2-Kuras 3
207.	D.IVPFY.F	637.3475	5	−0.3	638.3546	102	106	Patatin−2-Kuras 3
208.	L.AKSPEL.D	643.3541	6	−2.7	322.6834	171	176	Patatin−2-Kuras 3
209.	K.NGYPRL.V	718.3762	6	−0.7	360.1951	193	198	Putative Kunitz-type tuber invertase inhibitor
210.	F.AISTSKL.C	718.4225	7	−0.2	360.2184	118	124	Serine protease inhibitor 1
211.	F.HLVEPK.Y	721.4122	6	−3.5	361.7121	108	113	Probable Inactive Patatin−3-Kuras 1
212.	Y.FEHGPH.I	722.3136	6	−0.8	362.1638	107	112	Patatin−2-Kuras 3
213.	L.IGVPTKL.A	726.4639	7	0.0	364.2392	51	57	Proteinase inhibitor I
214.	Y.RIISTF.W	735.4279	6	−0.7	368.7210	51	56	Serine protease inhibitor 1
215.	L.SIDGGGIK.G	745.3970	8	−3.1	746.4019	33	40	Patatin−07
216.	L.AKSPELD.A	758.3810	7	−1.6	380.1971	171	177	Patatin−2-Kuras 3
217.	F.TKSNLAK.S	760.4443	7	−0.9	381.2291	166	172	Patatin−07
218.	K.LAQVDPK.F	769.4333	7	−2.7	385.7229	235	241	Patatin−2-Kuras 3
219.	L.LVQVGEK.L	771.4490	7	−1.0	386.7314	342	348	Patatin−2-Kuras 3
220.	L.VQVGEKL.L	771.4490	7	−0.1	386.7318	343	349	Patatin−2-Kuras 3
221.	R.AQEDPAF.A	776.3340	7	−0.4	777.3410	237	243	Patatin−01
222.	F.NIPTVKL.C	783.4854	7	0.9	392.7503	118	124	Kunitz-type protease inhibitor
223.	L.RIGERY.I	792.4242	6	−2.3	397.2184	51	56	Putative Kunitz-type tuber invertase inhibitor
224.	W.KVNHEGL.V	795.4239	7	−3.1	398.7180	141	147	Kunitz-type trypsin inhibitor
225.	R.VHQALTE.V	796.4079	7	0.6	797.4156	143	149	Patatin−07
226.	L.VKDNPLD.V	799.4075	7	−1.5	400.7104	208	214	Kunitz-type inhibitor B
227.	F.LGKGTPVM.F	801.4418	8	−2.4	401.7272	92	99	Putative Kunitz-type tuber invertase inhibitor
228.	F.AKLLSDR.K	801.4708	7	−3.8	401.7411	370	376	Patatin−07
229.	F.IPLSGGIF.E	802.4589	8	0.9	803.4655	72	79	Kunitz-type proteinase inhibitor group A1
230.	L.AQVDPKF.A	803.4177	7	−1.2	402.7157	236	242	Patatin−2-Kuras 3
231.	Y.IIKNPLL.G	809.5375	7	−1.3	405.7755	57	63	Putative Kunitz-type tuber invertase inhibitor
232.	Y.RIISIGR.G	813.5184	7	0.6	407.7662	26	32	Kunitz-type proteinase inhibitor group A1
233.	K.TNKPVIF.T	817.4698	7	−1.8	818.4756	159	165	Patatin−07

234.	L.LSVSVATR.R	831.4814	8	−1.4	416.7474	227	234	Patatin−07
235.	L.EFLEGQL.Q	834.4123	7	0.5	835.4200	49	55	Patatin−2-Kuras 3
236.	E.SPVPKPVL.D	835.5167	8	0.5	418.7654	34	41	Aspartic protease inhibitor 4
237.	A.VATVGDPAL.L	841.4545	9	−1.4	842.4606	218	226	Patatin−2-Kuras 3
238.	L.LAQVGENL.L	842.4498	8	−1.5	843.4558	343	350	Patatin−01
239.	W.KVNDEQL.V	844.4290	7	−3.1	423.2205	137	143	Putative Kunitz-type tuber invertase inhibitor
240.	E.SPLPKPVL.D	849.5323	8	−1.5	425.7728	34	41	Kunitz-type protease inhibitor
241.	L.LVQVGETL.L	857.4858	8	−1.6	429.7495	330	337	Patatin−13
242.	F.NLVDGAVAT.V	858.4447	9	−1.4	859.4508	212	220	Patatin−2-Kuras 3
243.	L.LVQVGENL.L	870.4811	8	1.6	871.4897	342	349	Patatin−07
244.	L.VTVDDDKD.F	905.3978	8	−2.7	453.7050	206	213	Cysteine protease inhibitor 1
245.	E.NALTGTTTK.A	905.4818	9	−3.7	453.7465	322	330	Patatin−07
246.	L.ISNVHILL.N	907.5491	8	−0.5	454.7816	33	40	Proteinase inhibitor I
247.	N.LPSDATPVL.D	911.4963	9	1.9	912.5053	29	37	Serine protease inhibitor 1
248.	N.LLKKPVSK.D	911.6168	8	−2.4	456.8145	349	356	Patatin−07
249.	K.LAQVDPKF.A	916.5018	8	−1.4	459.2575	235	242	Patatin−2-Kuras 3
250.	F.LGKGTPVVF.V	916.5381	9	−1.0	459.2759	98	106	Putative Kunitz-type tuber invertase inhibitor
251.	K.TNKPVIFT.K	918.5175	8	−3.3	460.2645	159	166	Patatin−07
252.	K.GIIPATILE.F	925.5484	9	−2.2	463.7805	29	37	Patatin−2-Kuras 1
253.	N.GKLSWPEL.I	928.5018	8	−1.9	465.2573	7	14	Proteinase inhibitor I
254.	F.GPMYDGKY.L	929.3953	8	−3.0	465.7035	110	117	Patatin−2-Kuras 1
255.	R.RAEEDPAF.A	933.4192	8	−1.9	467.7160	235	242	Patatin−07
256.	K.VGVVHQNGK.R	936.5141	9	−5.6	469.2617	194	202	Serine protease inhibitor 1
257.	F.AAAKDIIPF.Y	944.5331	9	−2.2	473.2728	97	105	Patatin−2-Kuras 2
258.	A.AKDIVPFY.F	951.5065	8	−0.5	476.7603	99	106	Patatin−07
259.	L.AKSPELDAK.M	957.5131	9	−3.9	479.7620	171	179	Patatin−07
260.	T.VLSIDGGGIK.G	957.5494	10	−2.5	479.7808	31	40	Patatin−07
261.	E.FLEGQLQK.M	961.5233	8	−3.2	481.7674	50	57	Patatin−01
262.	W.KVGNLNAHL.R	964.5454	9	0.0	322.5224	132	140	Kunitz-type protease inhibitor
263.	S.ESPVPKPVL.D	964.5593	9	0.7	483.2866	33	41	Aspartic protease inhibitor
264.	F.RYNSDVGR.S	965.4679	8	−4.0	483.7393	86	93	Kunitz-type protease inhibitor
265.	R.VHQALTEVA.I	966.5134	9	−0.9	484.2635	143	151	Patatin−07
266.	Y.LLQVLQEK.L	969.5859	8	0.0	485.8002	118	125	Patatin−2-Kuras 1
267.	S.ESPLPKPVL.D	978.5750	9	−2.8	490.2934	33	41	Kunitz-type protease inhibitor
268.	Y.FEHGPHIF.N	982.4661	8	−0.8	492.2399	107	114	Patatin−07
269.	Y.LMQVLQEK.L	987.5423	8	−2.7	494.7771	130	137	Patatin−07
270.	N.FKNGYPRL.V	993.5396	8	−3.6	497.7753	198	205	Cysteine protease inhibitor 1
271.	E.NALTGTATTF.D	995.4924	10	−3.1	996.4965	310	319	Probable Inactive Patatin−3-Kuras 1
272.	A.TKLAQVDPK.F	998.5760	9	−2.9	333.8650	233	241	Patatin−2-Kuras 3
273.	Y.FLQVLQEK.L	1003.5702	8	1.1	502.7929	130	137	Patatin−2-Kuras 3
274.	L.VNENPLDVL.F	1011.5237	9	−3.3	506.7675	208	216	Kunitz-type protease inhibitor
275.	D.VGPSGTPVRF.I	1015.5450	10	1.0	508.7803	87	96	Serine protease inhibitor 1
276.	F.IPLSTNIFE.N	1032.5491	9	0.5	1033.5569	101	109	Kunitz-type protease inhibitor
277.	Y.IINNPLLGAGA.V	1051.6025	11	−2.9	1.052.6068	63	73	Cysteine protease inhibitor 1
278.	Y.IIKNPLLGGGA.V	1051.6389	11	0.3	526.8269	61	71	Putative Kunitz-type tuber invertase inhibitor
279.	F.DVIGGTGTGGLL.T	1058.5608	12	1.8	1059.5699	59	70	Probable Inactive Patatin−3-Kuras 1
280.	D.IKTNKPVIF.T	1058.6488	9	−0.7	530.3313	157	165	Patatin−07
281.	A.KLEEMVTVL.S	1060.5839	9	0.0	531.2992	24	32	Patatin−07
282.	S.KVGVVHQNGK.R	1064.6090	10	−3.8	533.3098	193	202	Kunitz-type inhibitor B
283.	Y.IIKNPLLGAGA.V	1065.6545	11	−3.1	533.8329	57	67	Putative Kunitz-type tuber invertase inhibitor
284.	K.VGVVIQNGKR.R	1068.6404	10	−0.9	535.3270	194	203	Kunitz-type protease inhibitor
285.	F.FGPKYDGKY.L	1073.5182	9	−3.2	537.7646	122	130	Patatin−01
286.	E.FDKTHTAEE.T	1076.4774	9	−1.2	539.2454	267	275	Patatin−01
287.	L.DVAGKELDSR.L	1088.5461	10	−1.0	545.2798	38	47	Kunitz-type inhibitor B
288.	F.DVIGGTSTGGLL.T	1088.5713	12	1.0	545.2935	71	82	Patatin−07
289.	L.FPTSEGGLTGK.G	1092.5452	11	0.6	547.2802	846	856	Lipoxygenase OS=Solanum tuberosum
290.	K.VGVVHQNGKR.R	1092.6152	10	−3.4	547.3130	194	203	Serine protease inhibitor 1
291.	F.AAAKDIVPFY.F	1093.5807	10	−0.7	547.7972	97	106	Patatin−2-Kuras 3
292.	F.VTHTSNGARY.E	1104.5312	10	−4.9	369.1826	200	209	Patatin-B2
293.	F.AAAKDIIPFY.F	1107.5964	10	−0.6	554.8052	97	106	Patatin−2-Kuras 2
294.	L.LSLGTGTTSEF.D	1111.5397	11	−1.5	1112.5453	257	267	Patatin−01
295.	L.LVQVGENLLK.K	1111.6600	10	0.5	556.8376	342	351	Patatin−07
296.	L.DVTGKELDSR.L	1118.5568	10	−2.1	560.2845	38	47	Serine protease inhibitor 1
297.	F.VTHTSNGDKY.E	1120.5149	10	−3.0	561.2631	200	209	Patatin−2-Kuras 3
298.	L.LSLGTGTNSEF.D	1124.5349	11	−3.5	563.2728	256	266	Patatin−2-Kuras 3
299.	L.VQVGENLLKK.P	1126.6710	10	0.0	376.5643	343	352	Patatin−07
300.	L.VKDNPLDVSF.M	1132.5764	10	−1.6	567.2946	208	217	Kunitz-type inhibitor B

301.	A.TKLAQVDPKF.A	1145.6444	10	−0.5	382.8885	233	242	Patatin−2-Kuras 3
302.	Q.KMDNNADARL.A	1146.5452	10	−1.3	574.2791	57	66	Patatin−01
303.	A.TRLAQEDPAF.S	1146.5669	10	−2.2	574.2895	221	230	Patatin−2-Kuras 1
304.	L.VKDNPLDISF.K	1146.5920	10	−1.7	574.3023	208	217	Serine protease inhibitor 1
305.	Q.ILLNGSPVTKD.F	1155.6499	11	−0.4	578.8320	74	84	Proteinase inhibitor I
306.	Y.IIKNPLLGAGAV.Y	1164.7230	12	−1.8	583.3677	57	68	Putative Kunitz-type tuber invertase inhibitor
307.	L.VTVDDDKDFI.P	1165.5503	10	0.4	583.7827	206	215	Cysteine protease inhibitor 1
308.	R.VHQALTEVAIS.S	1166.6295	11	0.8	584.3225	143	153	Patatin−07
309.	F.DIKTNKPVIF.T	1173.6758	10	−1.8	587.8441	156	165	Patatin−07
310.	C.LKVGVVHQNGK.R	1177.6931	11	−2.5	393.5707	192	202	Kunitz-type inhibitor B
311.	K.SVSEDNHETY.E	1179.4680	10	−3.5	590.7392	341	350	Probable Inactive Patatin−3-Kuras 1
312.	L.VVTGGKVGNEND.I	1187.5782	12	−2.5	594.7949	150	161	Cysteine protease inhibitor 1
313.	L.DVAGKELDSRL.S	1201.6302	11	0.0	601.8224	38	48	Kunitz-type inhibitor B
314.	Y.FDVIGGTGTGGLL.T	1205.6292	13	−0.5	603.8215	58	70	Probable Inactive Patatin−3-Kuras 1
315.	T.VDDDKDFIPF.V	1209.5553	10	0.2	605.7848	208	217	Cysteine protease inhibitor 1
316.	F.QAHHSQNNYL.R	1210.5480	10	−0.3	606.2811	308	317	Patatin−2-Kuras 2
317.	S.KVGVVHQNGKR.R	1220.7102	11	0.9	306.1845	193	203	Kunitz-type inhibitor B
318.	L.DVTGKELDSRL.S	1231.6407	11	0.1	616.8276	38	48	Serine protease inhibitor 1
319.	L.LETGGTIGQADSS.W	1234.5677	13	0.4	618.2909	145	157	Serine protease inhibitor 1
320.	F.IGSSSHFGQGIF.E	1235.5935	12	0.8	618.8035	97	108	Serine protease inhibitor 1
321.	Y.FDVIGGTSTGGLL.T	1235.6398	13	−1.1	618.8265	70	82	Patatin−07
322.	L.LVQVGENLLKK.P	1239.7550	11	−1.1	414.2585	342	352	Patatin−07
323.	L.QEVDNNADARL.A	1243.5792	11	−1.0	622.7963	44	54	Patatin−2-Kuras 4
324.	A.LVKDNPLDVSF.M	1245.6604	11	−1.0	623.8369	207	217	Kunitz-type inhibitor B
325.	F.VRKSESDYGDV.V	1253.5887	11	−1.3	627.8002	107	117	Cysteine protease inhibitor 1
326.	R.KSESDYGDVVR.V	1253.5887	11	−2.3	627.8002	109	119	Cysteine protease inhibitor 1
327.	F.DVIGGTSTGGLLTA.M	1260.6561	14	0.5	1261.6641	71	84	Patatin−07
328.	K.SVSKDNPETYE.E	1267.5568	11	−0.1	634.7856	353	363	Patatin- T5
329.	L.QKMDNNADARL.A	1274.6038	11	−1.0	638.3085	56	66	Patatin−01
330.	L.DTNGKELNPDSS.Y	1275.5579	12	−0.7	638.7858	13	24	Kunitz-type proteinase inhibitor group A1
331.	A.VEDSSSPHGVRL.L	1281.6313	12	−1.3	428.2172	618	629	Lipoxygenase OS=Solanum tuberosum
332.	L.AIQQMTNAASSY.M	1283.5815	12	0.1	642.7981	274	285	Patatin−2-Kuras 1
333.	L.AFNPGHIVPGIY.Y	1283.6663	12	−1.5	642.8394	323	334	Putative Kunitz-type tuber invertase inhibitor
334.	L.QHMSIPQFLGK.G	1284.6648	11	−2.3	429.2279	84	94	Putative Kunitz-type tuber invertase inhibitor
335.	L.QEVDNNKDARL.A	1300.6371	11	−2.2	651.3244	56	66	Patatin−07
336.	M.ITTPNENNRPF.A	1301.6364	11	−2.0	651.8242	86	96	Patatin−07
337.	R.LAQEDPAFSSIK.S	1304.6611	12	0.3	653.3380	223	234	Patatin−2-Kuras 1
338.	K.SVSEDNHETYE.V	1308.5106	11	−1.0	655.2619	341	351	Probable Inactive Patatin−3-Kuras 1
339.	Y.IINNPLLGAGAVY.L	1313.7343	13	−0.4	657.8741	63	75	Cysteine protease inhibitor 1
340.	Y.IIKNPLLGGGAVY.L	1313.7706	13	0.0	657.8926	61	73	Putative Kunitz-type tuber invertase inhibitor
341.	R.KSESDDGDVVRL.M	1318.6365	12	−2.4	440.5517	103	114	Putative Kunitz-type tuber invertase inhibitor
342.	R.KTDLVTPEGSKF.V	1320.6925	12	−2.5	441.2370	165	176	Kunitz-type trypsin inhibitor
343.	L.LKKPVSKDSPET.Y	1327.7346	12	−3.1	664.8726	351	362	Patatin−13
344.	Y.IIKNPLLGAGAVY.L	1327.7864	13	−2.1	664.8990	57	69	Putative Kunitz-type tuber invertase inhibitor
345.	L.ALVKDNPLDISF.K	1330.7133	12	−2.6	666.3622	206	217	Serine protease inhibitor 1
346.	Y.NSDVGPSGTPVRF.I	1331.6470	13	−0.3	666.8306	84	96	Serine protease inhibitor 1
347.	Y.FDVIGGTSTGGLLT.A	1336.6874	14	0.3	669.3512	70	83	Patatin−07
348.	M.ILLVQVGENLLK.K	1337.8282	12	−0.2	669.9213	328	339	Probable Inactive Patatin−3-Kuras 1
349.	R.YNSDVGPSGTPVR.F	1347.6418	13	1.2	674.8290	83	95	Serine protease inhibitor 1
350.	F.RYNSDVGPSGTPV.R	1347.6418	13	0.0	674.8282	82	94	Serine protease inhibitor 1
351.	L.LKKPVSKDNPET.Y	1354.7456	12	2.4	678.3817	351	362	Patatin−01
352.	R.RAQEDPAFASIR.S	1359.6895	12	−3.4	454.2356	236	247	Patatin−01
353.	F.IGSSSHFGQGIFE.N	1364.6360	13	−2.9	683.3233	97	109	Serine protease inhibitor 1
354.	E.RYIINNPLLGAGA.V	1370.7670	13	−1.1	686.3900	61	73	Cysteine protease inhibitor 1
355.	M.ITTPNENNRPFA.A	1372.6735	12	−0.2	687.3439	86	97	Patatin−07
356.	L.LETGGTIGQADSSY.F	1397.6310	14	1.2	699.8236	145	158	Kunitz-type protease inhibitor
357.	D.YFDVIGGTSTGGLL.T	1398.7031	14	0.9	700.3594	69	82	Patatin−07
358.	R.VHQALTEVAISSF.D	1400.7300	13	−1.9	701.3709	143	155	Patatin−07
359.	L.PEVYDQDGNPLR.I	1401.6525	12	1.0	701.8342	46	57	Cysteine protease inhibitor 1
360.	Y.FDVIGGTSTGGLLTA.M	1407.7245	15	1.5	704.8705	70	84	Patatin−07
361.	L.VTVDDDKDFIPF.V	1409.6714	12	−2.0	705.8416	206	217	Cysteine protease inhibitor 1
362.	R.TTLGGSAEYPYPR.R	1410.6779	13	0.8	706.3467	222	234	Lipoxygenase OS=Solanum tuberosum
363.	F.NLVDGAVATVGDPAL.L	1410.7355	15	0.6	706.3754	200	214	Patatin−2-Kuras 1
364.	F.DKTHTAQETAKW.G	1414.6841	12	−0.9	472.5682	267	278	Patatin−07
365.	F.DKTHTAEETAKW.G	1415.6681	12	−1.1	472.8961	268	279	Patatin−01

366.	L.YEGGIKLPQGPLF.K	1417.7605	13	−1.1	709.8867	314	326	Lipoxygenase OS=Solanum tuberosum
367.	L.RVQENALTGTTTE.M	1418.7001	13	2.0	710.3588	306	318	Patatin−2-Kuras 1
368.	L.LETGGTIGQADSSW.F	1420.6470	14	0.6	711.3312	145	158	Serine protease inhibitor 1
369.	Y.STAAAPTYFPPHY.F	1421.6615	13	−0.1	711.8380	186	198	Patatin−07
370.	Y.IINNPLLGAGAVYL.Y	1426.8184	14	−1.0	714.4157	63	76	Cysteine protease inhibitor 1
371.	L.VTVHDDKDFIPF.V	1431.7034	12	−2.5	478.2405	203	214	Putative Kunitz-type tuber invertase inhibitor
372.	A.MITTPNENNRPF.A	1432.6769	12	−2.4	717.3440	85	96	Patatin−07
373.	L.VVTGGNVGNENDIF.K	1433.6787	14	1.6	717.8478	144	157	Putative Kunitz-type tuber invertase inhibitor
374.	K.KSVSEDNHETYE.V	1436.6056	12	−0.1	719.3100	340	351	Probable Inactive Patatin−3-Kuras 1
375.	Y.IIKNPLLGAGAVYL.D	1440.8704	14	4.5	721.4457	57	70	Kunitz-type proteinase inhibitor group A1
376.	R.LALVKDNPLDISF.K	1443.7972	13	0.7	722.9064	205	217	Kunitz-type inhibitor B
377.	L.VVTGGKVGNENDIF.K	1447.7307	14	1.5	724.8737	150	163	Cysteine protease inhibitor 1
378.	F.ANQPYLPSKTPEL.L	1456.7561	13	−1.7	729.3841	163	175	Lipoxygenase OS=Solanum tuberosum
379.	L.DVTGKELDSHLSY.R	1462.6940	13	0.3	488.5721	38	50	Kunitz-type inhibitor B
380.	L.LSLGTGTNSEFDKT.H	1468.7046	14	3.3	735.3620	256	269	Patatin−07
381.	T.VFQDLHSQNNYL.R	1476.6997	12	−3.0	739.3549	306	317	Patatin−07
382.	E.HIEDKLDGLTVDE.A	1482.7202	13	−1.5	742.3663	412	424	Lipoxygenase OS=Solanum tuberosum
383.	L.LKKPVSKDSPETY.E	1490.7980	13	−3.8	497.9380	351	363	Patatin−13
384.	E.NALTGTTTKADDASE.A	1493.6846	15	1.3	747.8505	322	336	Patatin−07
385.	R.YNSDVGPSGTPVRF.I	1494.7102	14	−1.0	748.3616	83	96	Serine protease inhibitor 1
386.	T.AMITTPNENNRPF.A	1503.7140	13	−1.7	752.8630	84	96	Patatin−07
387.	L.KKPVSKDSPETYE.E	1506.7566	13	−0.9	754.3849	352	364	Patatin−13
388.	F.VRKSESDYGDVVR.V	1508.7583	13	−2.9	503.9253	107	119	Cysteine protease inhibitor 1
389.	R.GVAVEDSSSPHGVRL.L	1508.7583	15	−2.1	503.9256	615	629	Lipoxygenase OS=Solanum tuberosum
390.	W.LLAIQQMTNAASSY.M	1509.7498	14	1.4	755.8832	272	285	Patatin−2-Kuras 1
391.	L.LKKPVSKDNPETY.E	1517.8090	13	−1.5	506.9428	351	363	Patatin−01
392.	L.SLGTGTNSEFDKTY.T	1518.6838	14	0.3	760.3494	245	258	Patatin−2-Kuras 1
393.	F.NLVDGAVATVGDPALL.S	1523.8195	16	2.3	762.9188	200	215	Patatin−2-Kuras 1
394.	Y.LLQVLQEKLGETR.V	1525.8828	13	−2.3	509.6337	118	130	Patatin−2-Kuras 1
395.	L.RVQENALTGTTTEL.D	1531.7842	14	−1.1	766.8985	318	331	Patatin-T5
396.	M.LLETGGTIGQADSSW.F	1533.7311	15	2.0	767.8743	144	158	Serine protease inhibitor 1
397.	L.SIDGGGIKGIIPAIIL.E	1535.9286	16	0.0	768.9716	33	48	Patatin−07
398.	F.NLVDGAVATVADPALL.S	1537.8351	16	−2.8	769.9227	212	227	Patatin−07
399.	Y.FVTHTSNGDKYEF.N	1543.6943	13	1.0	772.8552	199	211	Patatin−2-Kuras 3
400.	L.LETGGTIGQADSSYF.K	1544.6995	15	1.5	773.3582	145	159	Kunitz-type protease inhibitor
401.	Y.STAAAPIYFPPHHF.V	1554.7618	14	−3.2	519.2596	186	199	Patatin
402.	Y.FLQVLQEKLGETR.V	1559.8671	13	−1.4	520.9622	130	142	Patatin−2-Kuras 3
403.	E.FDKTHTAEETAKW.G	1562.7365	13	−1.4	521.9187	267	279	Patatin−01
404.	E.NALTGTTTKADDASEA.N	1564.7217	16	2.5	783.3701	322	337	Patatin−07
405.	W.MLVIQQMTEAASSY.M	1570.7371	14	−3.7	786.3729	284	297	Patatin−07
406.	N.LVLPEVYDQDGNPL.R	1570.7878	14	2.3	786.4030	43	56	Cysteine protease inhibitor 1
407.	R.LALVKDNPLDISFK.Q	1571.8922	14	1.5	786.9546	205	218	Kunitz-type inhibitor B
408.	F.VRKSESDDGDVVRL.M	1573.8059	14	−2.7	525.6078	101	114	Putative Kunitz-type tuber invertase inhibitor
409.	L.ISNVHILLNGSPVTL.D	1575.8984	15	−1.5	788.9553	33	47	Proteinase inhibitor I
410.	L.FIQTMDPEDVDKF.D	1583.7178	13	0.1	792.8663	270	282	Putative Kunitz-type tuber invertase inhibitor
411.	L.TAMITTPNENNRPF.A	1604.7617	14	0.2	803.3883	83	96	Patatin−07
412.	L.LKKPVSKDSPETYE.E	1619.8406	14	0.5	810.9280	351	364	Patatin−13
413.	L.LSLGTGTNSEFDKTY.T	1631.7678	15	1.6	816.8925	244	258	Patatin−2-Kuras 1
414.	L.LKKPVSKDNPETYE.E	1646.8514	14	−1.8	824.4315	350	363	Patatin−07
415.	F.RYNSDVGPSGTPVRF.I	1650.8114	15	0.3	826.4132	82	96	Serine protease inhibitor 1
416.	I.FRYNSDVGPSGTPVR.F	1650.8114	15	−1.7	551.2768	81	95	Kunitz-type inhibitor B
417.	E.FNLVDGGVATVGDPALL.S	1656.8722	17	−1.3	829.4423	211	227	Patatin group M−1
418.	Y.FVTHTSNGDKYEFN.L	1657.7372	14	−0.2	553.5862	199	212	Patatin−2-Kuras 3
419.	R.LADYFDVIGGTGTGGLL.T	1667.8406	17	1.2	834.9286	54	70	Probable Inactive Patatin−3-Kuras 1
420.	E.NALTGTTTKADDASEAN.M	1678.7645	17	0.8	840.3902	323	339	Patatin−02
421.	R.LADYFDVIGGTSTGGLL.T	1697.8512	17	0.3	849.9331	66	82	Patatin−07
422.	F.LEGQLQEVDNNKDAR.L	1727.8438	15	0.0	576.9552	51	65	Patatin−07
423.	F.QLISSVQGDPTNGLQGK.H	1740.9006	17	−0.4	871.4572	58	74	Lipoxygenase Solanum tuberosum
424.	L.LKKPVSKDSPETYEE.A	1748.8832	15	0.8	875.4496	351	365	Patatin−13
425.	L.LKKPVSKDNPETYEE.A	1775.8940	15	−0.6	888.9538	350	364	Patatin−07
426.	V.FRYNSDVGPSGTPVRF.I	1797.8798	16	0.4	600.3008	81	96	Serine protease inhibitor 1
427.	E.NALTGTTTKADDASEANM.E	1809.8051	18	1.0	905.9107	322	339	Patatin−07
428.	L.LKKPVSKDSPETYEEA.L	1819.9203	16	−1.6	910.9660	351	366	Patatin−13
429.	F.LEGQLQEVDNNKDARL.A	1840.9279	16	−0.3	614.6497	51	66	Patatin−07
430.	L.LKKPVSKDNPETYEEA.L	1846.9312	16	1.0	924.4738	350	365	Patatin−07
431.	R.VQENALTGTTTKADDASE.A	1849.8541	18	1.2	925.9354	319	336	Patatin−07
432.	K.SVSKDNPETYEEALKR.F	1864.9166	16	0.1	622.6462	353	368	Patatin-T5

433.	R.LADYFDVIGGTSTGGLLTA.M	1869.9359	19	−2.2	935.9731	66	84	Patatin−07
434.	E.FLEGQLQEVDNNKDAR.L	1874.9122	16	−2.2	625.9766	50	65	Patatin−07
435.	L.QHMSIPQFLGEGTPVVF.V	1885.9396	17	2.9	943.9798	90	106	Cysteine protease inhibitor 1
436.	R.LADYFDVIGGTGTGGLLTAM.I	1970.9659	20	−2.8	986.4875	54	73	Probable Inactive Patatin−3-Kuras 1
437.	E.FLEGQLQEVDNNKDARL.A	1987.9962	17	−4.5	995.0009	50	66	Patatin−07
438.	K.KPVSKDNPETYEEALKR.F	2003.0323	17	−3.7	501.7635	352	368	Patatin−07
439.	L.RVQENALTGTTTKADDASE.A	2005.9552	19	−2.3	669.6575	318	336	Patatin−07
440.	N.LPSDATPVLDVTGKELDSR.L	2012.0425	19	−0.4	671.6878	29	47	Kunitz-type inhibitor B
441.	W.SIRIPDVDSKPVIPHNSR.V	2029.1068	18	−2.0	508.2830	189	206	1,4-alpha-glucan-branching enzyme
442.	E.NALTGTTTKADDASEANMEL.L	2051.9316	20	2.0	1026.9751	322	341	Patatin−07
443.	E.NALTGTTTEMDDASEANMEL.L	2112.8828	20	−2.5	1057.4460	310	329	Patatin−2-Kuras 1
444.	L.EFLEGQLQEVDNNKDARL.A	2117.0388	18	0.4	706.6872	49	66	Patatin−07
445.	L.RVQENALTGTTTKADDASEAN.M	2191.0352	21	1.5	731.3534	318	338	Patatin−07
446.	L.LKKPVSKDSPETYEEALKR.F	2217.2004	19	−1.5	444.4467	350	368	Patatin−2-Kuras 3
447.	L.RVQENALTGTTTKADDASEANM.E	2322.0757	22	−0.4	775.0322	318	339	Patatin−07
448.	L.RVQENALTGTTTKADDASEANMEL.L	2564.2024	24	1.4	855.7426	318	341	Patatin−07
449.	F.DVIGGTSTGGLLTAMITTPNENNRPF.A	2675.3225	26	3.5	1338.6732	71	96	Patatin−11
450.	Y.LRVQENALTGTTTKADDASEANMEL.L	2677.2864	25	−0.1	893.4360	318	342	Patatin−01
